# The genes *zntA* and *zntR* encoding a P-type ATPase and its activator confer zinc and cadmium resistance in *Chromobacterium violaceum*

**DOI:** 10.1007/s10534-026-00828-4

**Published:** 2026-05-22

**Authors:** Carolina Panosso Schuindt, Bianca Bontempi Batista, Lucas Cesetti, Sergio Pereira Lima Neto, Thierry Soldati, José Freire da Silva Neto

**Affiliations:** 1 Departamento de Biologia Celular e Molecular e Bioagentes Patogênicos, Faculdade de Medicina de Ribeirão Preto, Universidade de São Paulo, Ribeirão Preto, Brazil; 2 Department of Biochemistry, University of Geneva, Sciences II, Geneva-4, CH-1211 Geneva, Switzerland

**Keywords:** *Chromobacterium violaceum*, Zinc resistance, Efflux pump, Transcription factor, Cadmium resistance, Metal toxicity

## Abstract

The transition metal zinc is an essential trace element for life but becomes toxic at high concentrations. In the opportunistic pathogen *Chromobacterium violaceum*, zinc acquisition is crucial for virulence, yet how this bacterium copes with zinc toxicity remains unknown. In this work, we demonstrate that *C. violaceum* senses and responds to excess zinc by activating the *zntA* gene, which encodes a P_1B-2_-type ATPase, via the transcriptional regulator ZntR. Phenotypic analyses by growth curves, disk diffusion, and CFU assays revealed that both Δ*zntA* or Δ*zntR* mutant strains exhibited increased sensitivity to zinc and cadmium. The Δ*zntA* mutant showed increased zinc and cadmium accumulation, suggesting that ZntA functions as an efflux pump. The expression of *zntA* increased in the presence of zinc and cadmium in the wild-type strain, but not in the Δ*zntR* mutant strain, indicating that ZntR activates *zntA* under conditions of zinc or cadmium excess. The role of ZntA in vivo was analyzed using the social amoeba *Dictyostelium discoideum* as a host cell. Plaque assays indicated that the *C. violaceum* Δ*zntA* mutant strain was more susceptible than the *C. violaceum* wild-type strain to clearance by *D. discoideum* knockout strains with altered intracellular zinc allocation. Overall, our data reveal that the genes *zntA* and *zntR* encoding a P_1B-2_-type ATPase and its transcriptional activator are key determinants of zinc and cadmium resistance in *C. violaceum*, ensuring bacterial survival under zinc-stress conditions encountered in the environment and the host.

**Supplementary Information:**

The online version contains supplementary material available at 10.1007/s10534-026-00828-4.

## Introduction

Zinc is a crucial transition metal for living organisms, functioning as an essential cofactor for numerous proteins involved in key cellular processes, including signal transduction, metabolism, cell growth and division, transcription, and translation (Andreini et al. 2006; Remick and Helmann 2023). Despite its essentiality, zinc becomes toxic in excess due to protein mismetallation, in which zinc inappropriately binds to non-specific metalloproteins, disrupting key biochemical pathways (Capdevila et al. 2016; Chandrangsu et al. 2017). For instance, it has been shown in bacteria that zinc excess impairs oxidative stress resistance and manganese uptake (Eijkelkamp et al. 2014), causes heme accumulation and toxicity (Chandrangsu and Helmann 2016), and inhibits biofilm formation (Pan et al. 2023). In contrast, cadmium is a non-essential metal with virtually no known biological function that exhibits high toxicity to bacterial cells even in low doses (Begg et al. 2015; Neville et al. 2020; Remick and Helmann 2023).

Bacteria have evolved multiple strategies to handle excess metal ions, with efflux serving as the main mechanism of metal detoxification. Three major classes of transport systems are primarily involved in metal efflux: the P-type ATPases, the Cation Diffusion Facilitator (CDF) family transporters, and the Resistance-Nodulation-Division (RND)-type efflux pumps (Kolaj-Robin et al. 2015; Capdevila et al. 2016; Chandrangsu et al. 2017). The subfamily P_1B_ of P-type ATPases mediate the efflux of transition and heavy metals like Cu^+^, Zn^2+^, Co^2+^, Cd^2+^, and Pb^2+^ from the cytosol through the cell membrane. P_1B_-type ATPases are classified into subgroups based on metal specificity, with the P_1B-2_ subgroup specialized in zinc and cadmium efflux (Argüello 2003; Argüello et al. 2011; Rosenzweig and Argüello 2012; Smith et al. 2014; Sitsel et al. 2015). Although some bacterial P_1B-2_ proteins have been characterized as zinc and/or cadmium exporters, including ZntA (Rensing et al. 1997; Maunders et al. 2022; Zheng et al. 2024), CadA (Ducret et al. 2020), and CtpC (Botella et al. 2011; Hanna et al. 2021), for most bacteria their role remains unknown.

Bacterial transcriptional regulators that act as cytosolic zinc sensors coordinate the expression of zinc uptake and efflux systems. While the zinc uptake regulator Zur functions as a repressor that allows the expression of zinc uptake systems under zinc limitation, the MerR-family transcriptional regulator ZntR becomes critical under zinc excess (Capdevila et al. 2016; Chandrangsu et al. 2017). In *Escherichia coli*, ZntR senses zinc ions and activates expression of the zinc exporter ZntA by remodeling the *zntA* promoter, which has an extended 19–20 bp spacer between the –35 and –10 elements, typical of MerR-activated promoters (Rensing et al. 1997; Brocklehurst et al. 1999; Philips et al. 2015; Chandrangsu et al. 2017).

While zinc limitation imposed by host proteins such as calprotectin is a well-established aspect of nutritional immunity, zinc intoxication has also emerged as an additional host defense strategy against pathogens (Hood and Skaar 2012; Chandrangsu et al. 2017). During phagocytosis, phagocytic cells like macrophages and protozoa mobilize zinc into phagosomes, in part through fusion with zinc-containing vesicles (zincosomes), where elevated zinc levels contribute to microbial killing. As a countermeasure, bacterial pathogens such as *Salmonella enterica* serovar Typhimurium, uropathogenic *Escherichia coli* (UPEC), *Mycobacterium tuberculosis*, and *Mycobacterium marinum* express zinc efflux systems, including the P-type ATPases ZntA and CtpC (Kapetanovic et al. 2016; Stocks et al. 2019; Botella et al. 2011; Hanna et al. 2021). In *Dictyostelium discoideum*, a social amoeba with cell-autonomous defense mechanisms conserved in humans, four Zn^2^⁺ efflux transporters of the ZnT (Zn^2+^ transporter Slc30a) family have been identified. Among these, the ZnT proteins ZntA and ZntB (here referred to as DdZntA and DdZntB to avoid ambiguity with the unrelated ZntA of *C. violaceum*) play a central role in intracellular zinc homeostasis and amoeba resistance to bacterial infection (Barisch et al. 2018; Hanna et al. 2021).

*Chromobacterium violaceum* is a Gram-negative, rod-shaped, motile, facultative anaerobic bacterium that inhabits soil and water in tropical and subtropical regions (Vasconcelos et al. 2003; Batista and da Silva Neto 2017). *C. violaceum* can cause rare but severe infections in humans, spreading to multiple organs after entering the body through skin lesions (Batista and da Silva Neto 2017). To infect mammalian cells, such as macrophages and hepatocytes, *C. violaceum* relies on a Cpi1/1a type III secretion system (T3SS) (Miki et al. 2010; Batista and da Silva Neto 2017). The host innate immune response, through processes like pyroptosis and granuloma formation, plays a crucial role in containing *C. violaceum* infection (Maltez et al. 2015; Harvest et al. 2023). It has previously been shown that uptake systems for the metals iron and zinc are essential for *C. violaceum* virulence (Batista et al. 2019; Santos et al. 2020, 2021; de Lima et al. 2022). Particularly for zinc, ZnuABC has been characterized as a high-affinity ABC transporter required for zinc uptake and growth under zinc limitation (Santos et al. 2021), but systems for zinc efflux have not yet been investigated in *C. violaceum*. Using mutant strains and expression assays, we demonstrated here that the P-type ATPase ZntA and its activator ZntR protect *C. violaceum* against zinc and cadmium toxicity. A role of ZntA for *C. violaceum* defense against bacterial clearance was observed in vivo in *D. discoideum*, mainly in knockout amoeba strains with altered intracellular zinc allocation.

## Materials and methods

### Bacterial strains, plasmids, and growth conditions

The bacterial strains and plasmids used in this work are indicated in Table 1. *E. coli* and *C. violaceum* strains were cultured in Luria–Bertani (LB) medium at 37 °C. The cultures were supplemented, when appropriated, with kanamycin (50 μg/mL), tetracycline (10 µg/mL), ampicillin (100 μg/mL), or different metals (Sigma-Aldrich) as stated in the figures.

**Table 1:** Bacterial and amoeba strains and plasmids

Strain or Plasmid	Description^a^	Reference or source
*Escherichia coli*		
DH5α	Strain for cloning purpose	(Hanahan 1983)
S17-1	Strain for plasmid mobilization	(Simon et al. 1983)
*Chromobacterium violaceum*		
WT	*C. violaceum* ATCC 12472 WT strain	(Vasconcelos et al. 2003)
WT[mRFP1]	WT control expressing the mRFP1 fluorescent protein	This work
WT[pSEVA]	WT control strain harboring the empty pSEVA221 plasmid	This work
WT[p*zntA*-*lacZ*]	WT strain with *zntA* (CV_1154)-*lacZ* fusion	This work
Δ*zntA*	WT strain with *zntA* (CV_1154) gene deleted	This work
Δ*zntA*[pSEVA]	Δ*zntA* mutant with empty pSEVA221 plasmid	This work
Δ*zntA*[*zntA*]	Δ*zntA* mutant complemented with WT copy of *zntA* in the pSEVA221 vector	This work
Δ*zntA*[p*zntA*-*lacZ*]	Δ*zntA* strain with *zntA* (CV_1154)-*lacZ* fusion	This work
Δ*zntR*	WT strain with *zntR* (CV_1153) gene deleted	This work
Δ*zntR*[pSEVA]	Δ*zntR* mutant with empty pSEVA221 plasmid	This work
Δ*zntR*[*zntR*]	Δ*zntR* mutant complemented with WT copy of *zntR* in the pSEVA221 vector	This work
Δ*zntR*[p*zntA*-*lacZ*]	Δ*zntR* strain with *zntA* (CV_1154)-*lacZ* fusion	This work
Δ*zntR*[*zntR*][p*zntA*-*lacZ*]	Δ*zntR*[*zntR*] strain with *zntA*-*lacZ* fusion	This work
*Dictyostelium discoideum*		
Ax2 (Ka)	Wild-type strain, parental of *zntA* KO	Soldati Lab Collection
Ax2 (Ka) *zntA* KO	Wild-type strain Ax2 (Ka) knockout for *zntA* gene	(Barisch et al. 2018)
Ax4	Wild-type strain, parental of *zntB* KO	Soldati Lab Collection
Ax4 *zntB* KO	Wild-type strain Ax4 knockout for *zntB* gene	REMI library Prof. Christopher Thompson
Plasmids		
pNPTS138	Suicide vector containing *oriT*, *npt*, *sacB*; Kan^R^	M.R.K. Alley
pGEM-T-Easy	Cloning plasmid; Amp^R^	Promega
pRK*lacZ*290	pRK2-derived vector with promoterless *lacZ* gene; Tet^R^	(Gober and Shapiro 1992)
pSEVA221	Broad-host-range vector containing *oriT*, *oriRK2*; Kan^R^	(Silva-Rocha et al. 2013)
pBBR1-MCS5	mRFP1 expressed under the control of two constitutive P*lac* promoters in tandem cloned into the pBBR1-MCS2 vector; Kan^R^	(Bayer-Santos et al. 2019)

^a^ Kan, kanamycin; Tet, tetracycline; Amp, ampicillin; R, resistance

### Construction of *C. violaceum* null-mutant and complemented strains

Null-mutant strains were generated by a previously established allelic exchange mutagenesis protocol, using the suicide vector pNPTS138 and counter-selection on LB sucrose plates (Batista et al. 2019; Santos et al. 2021; de Lima et al. 2022). Null-mutant strains were derived from the wild-type (WT) strain ATCC 12472. Briefly, flanking regions of the target genes were amplified by PCR using Phusion DNA polymerase (Thermo Scientific), digested with specific restriction enzymes (Thermo Scientific), ligated at 16 °C with T4 DNA ligase (New England Biolabs), and cloned into pNPTS138. The constructs were introduced into *C. violaceum* via conjugation with *E. coli* strain S17-1. Counter-selection was performed on LB plates containing 16% sucrose. Primers used for cloning, sequencing, and mutant confirmation are listed in Table S1. For complementation of the null mutant strains, the corresponding genes containing their regulatory and coding regions were amplified by PCR with specific primers (Table S1) and cloned into the replicative plasmid pSEVA221. The constructions were introduced in the mutant strains by conjugation (Batista et al. 2019; Santos et al. 2021; de Lima et al. 2022).

### Growth curves

Overnight cultures of *C. violaceum* WT, mutant, and complemented strains were diluted to an optical density at 600 nm (OD_600nm_) of 0.01 in LB. The inocula were untreated or treated with metals to a final volume of 150 µL in 96-well plates. Cultures were grown aerobically with shaking at 37 °C in a BioTek Epoch 2 microplate reader (Agilent). Growth was monitored by measuring OD_600nm_ every 60 min. Experiments were performed with three biological replicates. Treatment conditions are indicated in the respective figures.

### Cell viability by counting CFU (colony-forming unit)

Overnight cultures of *C. violaceum* WT, mutant, and complemented strains were diluted to OD_600nm_ 0.01 in LB and grown with shaking at 37 °C until OD_600nm_ of 0.5–0.6. Cultures were untreated or treated with 1 mM ZnCl_2_ or 300 μM CdCl_2_ for 2 h with agitation at 37 °C. Serial dilutions were prepared in phosphate-buffered saline (PBS), and 10 µL aliquots were spotted onto LB agar plates. The number of CFUs was counted after plate incubation for 24 h at 37 °C (Santos et al. 2021). All experiments were performed in three independent biological replicates.

### Disk diffusion assays

Overnight cultures of *C. violaceum* WT, mutant, and complemented strains were diluted to an OD_600nm_ of 0.1 in LB in a final volume of 600 µL and spread onto LB agar plates. Four sterile 6-mm Whatman filter paper disks were placed onto the surface of the bacterial lawns. Then, 10 µL of increasing concentrations of the tested metal solutions were applied to the disks, along with a control disk containing sterile ultrapure water. Plates were incubated at 37 °C for 24 h, and the diameters of the inhibition halos were measured (Santos et al. 2021). All experiments were performed in three independent biological replicates. Treatment conditions are indicated in the respective figures.

### Cellular metal ion content analysis

Quantification of metal content was performed by inductively coupled plasma mass spectrometry (ICP-MS) as previously described (Chaoprasid et al. 2015; Maunders et al. 2022). Briefly, overnight cultures of *C. violaceum* WT, Δ*zntA* mutant, and Δ*zntA*[*zntA*] complemented strains were diluted to an OD_600nm_ of 0.01. Cultures were grown in biological triplicate to mid-log-phase (OD_600nm_ 0.8–1) in LB untreated or treated with 100 µM ZnCl_2_. After centrifugation, bacterial cells were washed three times with PBS containing 5 mM ethylenediaminetetraacetic acid (EDTA), and twice with PBS. Samples were weighed and dissolved in 50% (v/v) HNO_3_. The zinc and cadmium content in the cell pellet was determined using a NexION 2000 ICP mass spectrometer (PerkinElmer), and was expressed as micrograms of metal per gram of bacterial cells (µg/g wet weight).

### Transcriptional *lacZ* fusions and β-galactosidase assays

The upstream region of the *zntA* gene was amplified by PCR using specific primers (Table S1). The *zntA* promoter DNA fragment was first cloned into the pGEM-T Easy vector (Promega), and subsequently subcloned into the pRK*lacZ*290 reporter plasmid. *C. violaceum* strains harboring the reporter plasmid were grown in LB medium at 37 °C to an OD_600nm_ of 0.5, and cultures were untreated or treated with metals or metal chelators for 45 min, when using *znt* mutant strains, or for 2 h, when using the WT strain. Bacterial cells were assayed for β-galactosidase activity as previously described (Santos et al. 2020, 2021; de Lima et al. 2022). The experiment was performed in three biological replicates.

### Gene expression by RT-qPCR

*C. violaceum* WT, Δ*zntA* mutant, and Δ*zntA*[*zntA*] complemented strains were grown in LB to OD_600nm_ 0.8–1.0, and the cultures were either untreated or treated with 250 µM ZnCl_2_ for 15 min. Total RNA was extracted using Trizol reagent (Invitrogen) and purified with Direct-zol RNA Miniprep Plus (Zymo Research). RNA from each sample was converted to cDNA using the High-Capacity cDNA Reverse Transcription kit (Thermo Fisher Scientific). Quantitative PCR (qPCR) reactions were performed on a QuantStudio 3 Real-Time PCR System (Thermo Fisher Scientific) using PowerUp SYBR Green Master Mix (Thermo Fisher Scientific), specific primers for *zntA* (Table S1), and cDNA templates. Data from three biological replicates were normalized by an endogenous control (gene CV_3376–*minD*) and a reference condition (WT strain in LB).

### *D. discoideum* strains and culture

The *D. discoideum* strains used in this study are listed in Table 1. The *D. discoideum* strains were cultured in autoclaved HL5c medium (Formedium) supplemented with 100 U/mL penicillin and 100 μg/mL streptomycin. Adherent cultures were incubated in Petri dishes at 22 °C in a lighted incubator and passaged every 2 days (Barisch et al. 2018).

### Generation of a fluorescent *C. violaceum* strain

A fluorescent *C. violaceum* strain was generated by introducing a stably replicating pBBR1-MCS2 (Km^R^) plasmid carrying a gene that constitutively expresses the mRFP1 fluorescent protein. The plasmid was first introduced into the conjugative *E. coli* S17-1 by transformation, and subsequently transferred to the *C. violaceum* WT strain via conjugation. Positive colonies were selected on kanamycin plates, and fluorescence was confirmed by confocal microscopy.

### Intracellular killing of bacteria assessed by live microscopy

An overnight culture of *C. violaceum* expressing mRFP1 was diluted to a density of 1.5 × 10^6^ cells/mL in sterile Sörensen Phosphate Buffer. Then, 200 μL of the normalized culture was added to individual wells of a μ-Slide 8-Well plate (glass bottom, IBIDI) previously coated overnight with 250 μL of 1 mg/mL Poly-L-Lysine (Sigma-Aldrich). The slides were centrifuged at room temperature for 10 min at 4,500 rpm. Immediately before imaging, 200 μL of *D. discoideum* Ax2 (Ka) at a density of 5 × 10^5^ cells/mL in filtered HL5c medium was added to each well. Bacterial cells were visualized using the mRFP1 fluorescence channel, while amoebae were imaged using bright-field microscopy. Time-lapse images were acquired at 30-s intervals for 2 h using a spinning disk confocal microscope at the Bioimaging Center of the Faculty of Science of the University of Geneva. Image processing was performed in Fiji (ImageJ), including scale bar insertion, background subtraction, and application of color codes. Fluorescence background was corrected using non-fluorescent *D. discoideum* Ax2 (wild type) cells as a reference at each time point. Individual contacts between amoebae and bacteria were quantified, recording for each bacterium the duration of contact, whether phagocytosis occurred, and the time of mRFP1 loss. In total, five independent videos were acquired, analyzing 50 *D. discoideum* cells and 295 *C. violaceum* cells.

### Infection of *D. discoideum* with *C. violaceum*

An overnight culture of *C. violaceum* expressing mRFP1 was centrifuged at room temperature for 10 min at 4500 rpm, and the pellet was resuspended in HL5c medium. The OD_620nm_ was measured, and the culture was adjusted to a multiplicity of infection (MOI) of 10 or 20. A confluent 10-cm dish of *D. discoideum* cells grown in 10 mL of HL5c medium was used for infection. The medium was removed and replaced with 5 mL of fresh HL5c. Subsequently, 500 μL of the bacterial suspension at MOI 10 or 20 was added to the adherent amoebae, followed by two centrifugation steps at 1,500 rpm for 10 min each to promote contact. The cells were incubated at room temperature for an additional 20 min to allow phagocytosis. Extracellular bacteria were eliminated by washing the cells seven times with 7 mL of HL5c supplemented with 60 μg/mL gentamicin. The infected cells were then resuspended in 10 mL of filtered HL5c containing gentamicin and diluted to a final density of 1.3 × 10^6^ cells/mL. The suspension was transferred to an IBIDI μ-Slide for imaging. Infected cells were imaged using a spinning-disk confocal microscope at the Bioimaging Center of the Faculty of Science of the University of Geneva after 45 min and 16 h post-infection (hpi). Image processing was performed in Fiji (ImageJ), including scale bar insertion, background subtraction, and application of color codes.

### Plaque assay

Overnight cultures of *C. violaceum* WT and *zntA* mutant strains were adjusted to an OD_600nm_ of 3.0 and diluted 1:100 in LB medium. Then, 50 µL of the diluted bacterial cultures were added to each well of a 24-well plate, each well containing 2 mL of SM-agar medium (10 g/L glucose, 10 g/L peptone, 1 g/L yeast extract, 1 g/L MgSO_4_·7H_2_O, 1.9 g/L KH_2_PO_4_, 20 g/L Bacto Agar, pH 6.0—6.4), and allowed to dry for 1 h. *D. discoideum* cells were prepared at different densities (5,000; 2,500; 1,250; and 650 cells per 5 µL). Subsequently, 5 µL of amoeba suspensions were spotted in the center of each well. Plates were incubated at 22 °C and images were taken over a period of 7 days. The assays were performed three times independently, and the images shown are representative of the experiment.

### Statistical analysis

Data collected were employed for statistical analysis in GraphPad Prism version 7. Most experiments were analyzed by two-way ANOVA followed by Tukey’s post hoc test. The β-galactosidase assay was analyzed using two-way ANOVA followed by Sidak’s multiple comparisons test. Nonparametric alternatives were applied when appropriate. All assays were performed with three biological replicates, and error bars represent the standard deviation (SD). Statistically significant p values and the tests that were performed are indicated in the figure legends.

## Results

### *zntA* encodes a P_1B-2_-type ATPase, and its expression responds specifically to zinc and cadmium

**Fig. 1:**
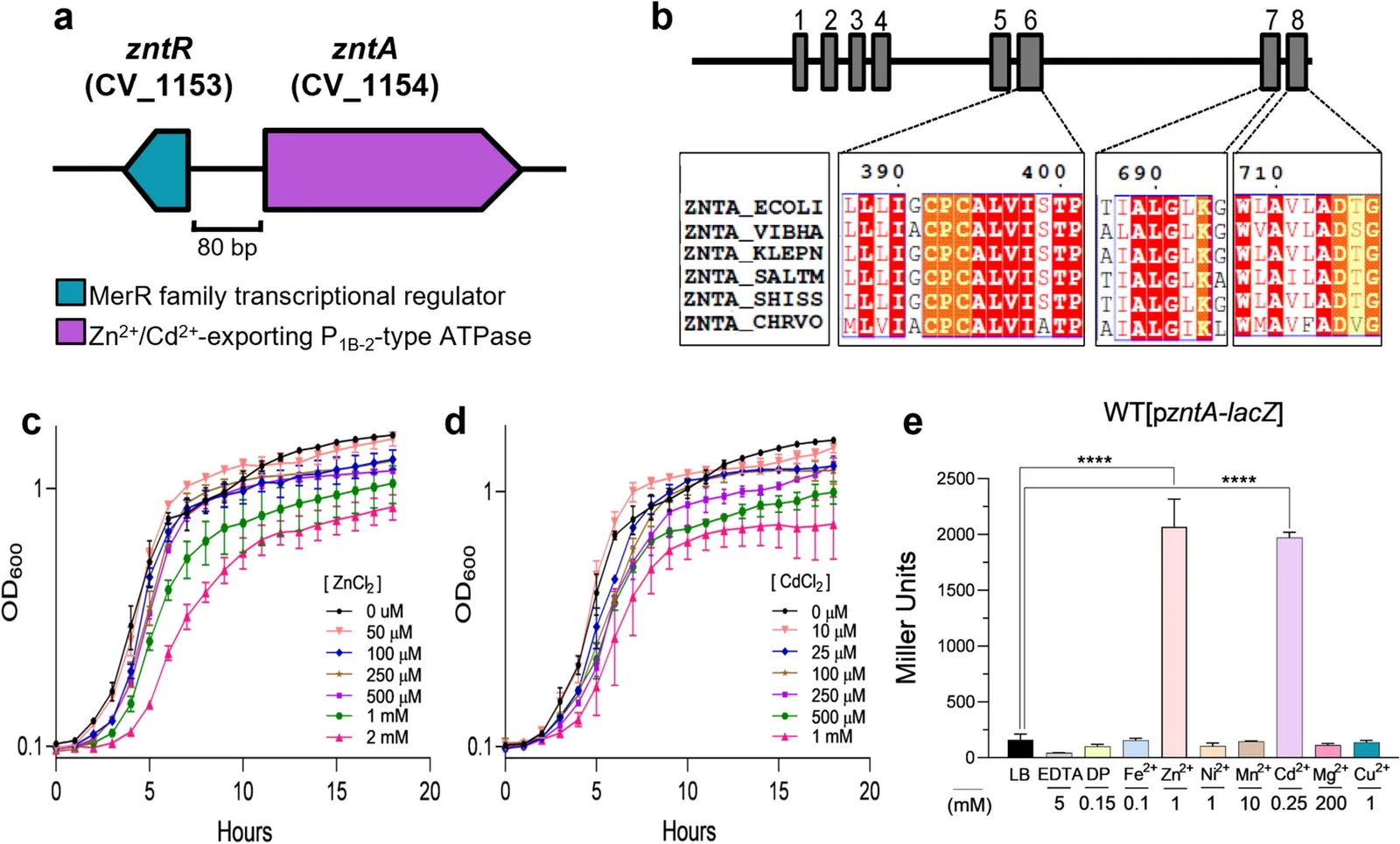
Genomic organization, P-type ATPase classification, and expression of *zntA* in *C. violaceum*. **a** Genomic organization of *zntA* showing the divergently transcribed gene *zntR.* **b** In silico analysis classifies ZntA as a P_1B-2_-type ATPase. Gray boxes indicate transmembrane helices; red highlights metal-selectivity amino acid residues; yellow marks zinc-specific signature residues. Multiple alignment used ZntA proteins from *C. violaceum* (CHRVO; Q7NYW8), *E. coli* (ECOLI; P37617), *V. harveyi* (VIBHA; A0A0D0HJV8), *K. pneumoniae* (KLEPN; W9BMV3), *S. typhimurium* (SALTM; Q8ZLE5), and *S. sonnei* (SHISS; Q3YW59). **c-d** Growth curves of *C. violaceum* wild type (WT) in LB with increasing concentrations of ZnCl_2_ (**c**) or CdCl_2_ (**d**). Data are means of three independent experiments. **e** *zntA* promoter activity increases in response to zinc and cadmium, but not to other metals or chelators. β-galactosidase activity assays were performed in *C. violaceum* WT carrying a p*zntA-lacZ* fusion grown in LB or LB with EDTA (5 mM), DP (150 μM), FeSO_4_ (100 μM), ZnCl_2_ (1 mM), NiSO_4_ (1 mM), MnCl_2_ (10 mM), CdCl_2_ (250 μM), MgSO_4_ (200 mM), or CuSO_4_ (1 mM), after 2 h treatment. Bars represent the mean ± SD of three biological replicates. Statistical significance was assessed by Two-Way ANOVA with Sidak’s test; ****p < 0.0001; when not indicated, not significant

To find determinants of zinc resistance in *C. violaceum*, a search in its genome (Vasconcelos et al. 2003) was performed for homologs of the P-type ATPase ZntA. A BlastP search using the *E. coli* ZntA sequence (Rensing et al. 1997) identified seven candidate proteins (Table S2), with the strongest and reverse best match corresponding to the product of gene CV_1154 (E-value: 10^–127^). The adjacent and divergently transcribed gene CV_1153 encodes a MerR family transcriptional regulator (Fig. 1a). Based on the functional characterization presented in this study, CV_1154 was designated as *zntA* and CV_1153 as *zntR* (Fig. 1a). Multiple sequence alignment with ZntA proteins of other bacteria revealed that *C. violaceum* ZntA contains within the transmembrane helices (TM) 6, 7, and 8, the typical conserved signature amino acids of the P_1B-2_ subgroup of P-type ATPases, namely CPC in TM6, a K in TM7, and the DXG motif in TM8 (Fig. 1b) (Argüello 2003; Rosenzweig and Argüello 2012; Wang et al. 2014; Sitsel et al. 2015). A phylogenetic tree of selected characterized ZntA proteins recapitulates the expected relationships among beta and gamma proteobacteria. Notably, the arrangement of *zntA* and *zntR* as adjacent, divergently oriented genes was observed only in *C. violaceum* among the bacteria analyzed (Fig. S1).

To investigate the resistance of *C. violaceum* to excess zinc and cadmium, growth curves were performed in Luria–Bertani (LB) medium supplemented with increasing concentrations of ZnCl_2_ (up to 2 mM) and CdCl_2_ (up to 1 mM) (Fig. 1c, d; Fig. S2). *C. violaceum* exhibited high tolerance to both metals, although cadmium was, as expected, more toxic than zinc. To assess the expression profile of *zntA* in response to zinc, cadmium, and other metals, β-galactosidase assays were performed in the *C. violaceum* WT strain using a *lacZ* reporter under the control of the *zntA* promoter. Exposure to excess metals strongly increased *zntA* promoter activity in the presence of zinc and cadmium, but not in the presence of the other metals tested (Fig. 1e). The metal chelator 2,2′-dipyridyl (DP) showed no effect, while the broad-spectrum metal chelator EDTA caused a slight, but not statistically significant, reduction in *zntA* expression compared to the LB medium condition (Fig. 1e). Taken together, these data indicate that the expression of *zntA* in *C. violaceum* is specifically upregulated in response to elevated levels of zinc and cadmium.

### *zntA* protects *C. violaceum* against zinc and cadmium toxicity

To address the role of ZntA in *C. violaceum* defense against metal toxicity, a Δ*zntA* null mutant strain and a Δ*zntA*[*zntA*] complemented strain were generated, and their growth and survival were compared with that of the WT strain. These three strains showed similar growth when cultured in LB medium under non-stress conditions (Fig. S3). However, in LB supplemented with high concentrations of copper, zinc, nickel, cadmium or manganese, the Δ*zntA* mutant exhibited growth impairment specifically under high zinc (1 mM) and cadmium (0.5 mM) conditions, whereas its growth in the presence of other metals was comparable to that of the WT strain (Fig. S3a–e). Additionally, in a disk diffusion assay under conditions of iron excess, the inhibition halo of the Δ*zntA* mutant was similar to that of the WT strain (Fig. S3f). These data indicate that ZntA is specifically involved in conferring protection against zinc and cadmium excess.

**Fig. 2:**
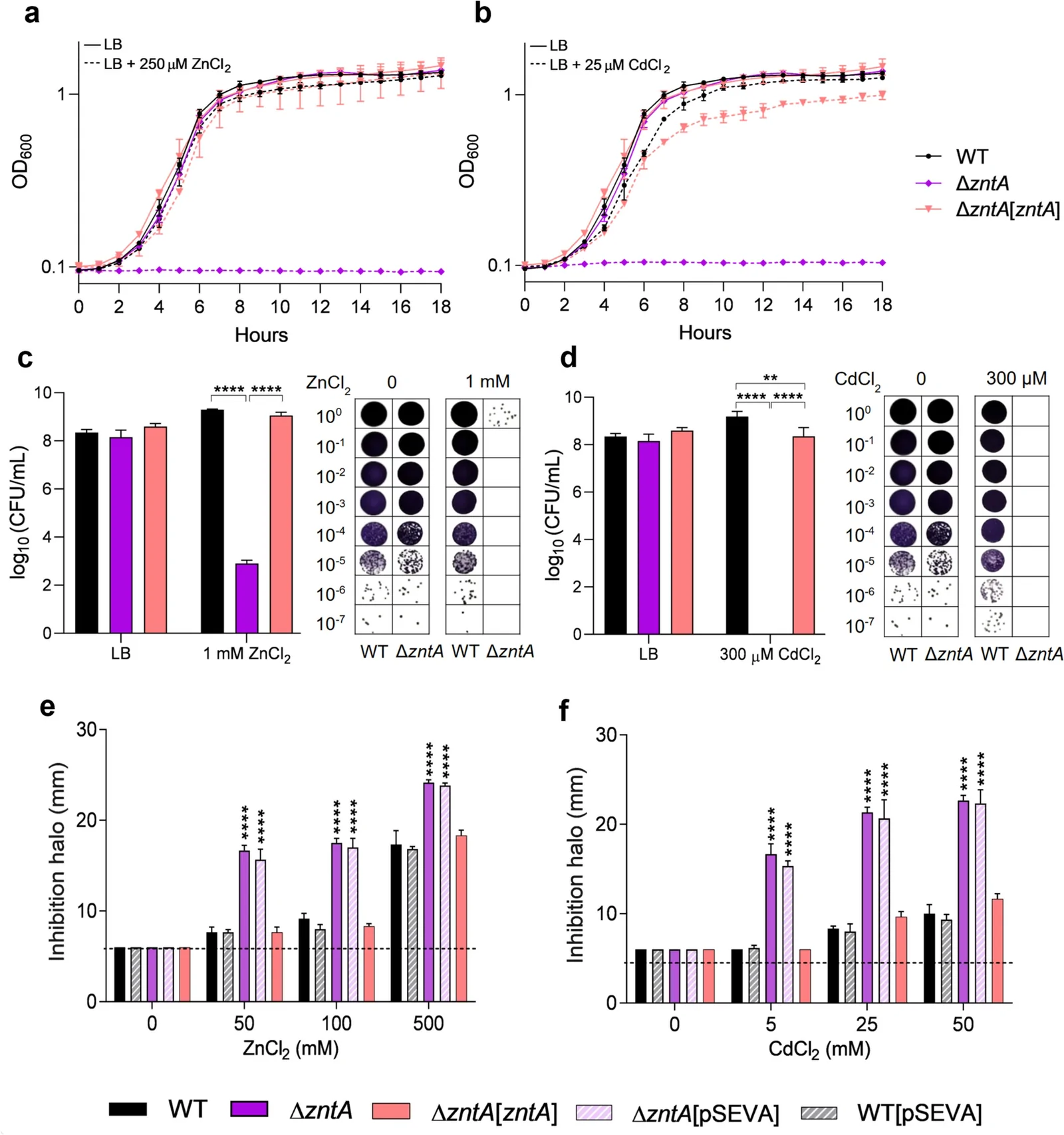
The *C. violaceum* Δ*zntA* mutant exhibits impaired growth and viability under zinc and cadmium excess. The indicated strains were grown in LB medium (solid lines) or LB plus ZnCl_2_ (**a**) or CdCl_2_ (**b**) (dashed lines). Bacterial growth was monitored using a BioTek Epoch 2 microplate reader, with OD_600nm_ readings recorded every 60 min over 18 h. Data represents the mean ± SD of three biological replicates. **c–d** Viability of the indicated *C. violaceum* strains was evaluated by counting of colony-forming units (CFU/ml). Bacterial cultures grown in LB until midlog growth phase were untreated (LB) or treated with 1 mM ZnCl_2_ (**c**) or 300 µM CdCl_2_ (**d**) for 2 h. Tenfold serial dilutions (10^0^ to 10⁻⁷ dilutions) were platted on LB for CFU counting after 24 h of incubation at 37 °C (representative images are shown). **e–f** Measurement of the growth inhibition halo diameter of the indicated strains in disk diffusion assays. Bacterial cultures were spread on the surface of LB plates; over the bacterial lawns were added paper disks without (0) or with increasing amounts of ZnCl_2_ (**e**) or CdCl_2_ (**f**). The dashed line indicates the disk size. Bars represent the mean ± SD of three independent biological experiments. Statistical significance was assessed by Two-Way ANOVA followed by Tukey’s multiple comparison test. Vertical bars indicate comparisons with the control condition (WT strain). **p < 0.01; ****p < 0.0001; when not indicated, not significant

To further examine this effect, growth-curve assays were performed in LB across four concentrations of zinc and cadmium (Fig. 2a, b; Fig. S4). For zinc, the Δ*zntA* mutant strain displayed complete growth inhibition at 250 µM (Fig. 2a) and gradual growth impairment in the other ZnCl_2_ concentrations (Fig. S4a, b, c). Under cadmium stress, the Δ*zntA* mutant showed greater sensitivity compared to zinc. Complete growth inhibition occurred at 25 µM (Fig. 2b), and the growth impairment occurred in a concentration-dependent manner (Fig. S4d, e, f). Genetic complementation fully restored Δ*zntA* growth in zinc and partially restored its growth in cadmium, maybe due to differences in *zntA* levels expressed from the plasmid (Fig. 2a, b; Fig. S4). The sensitivity of the Δ*zntA* mutant to zinc and cadmium was further confirmed by CFU counting (Fig. 2c, d) and disk diffusion assays (Fig. 2e, f). Indeed, upon exposure to 1 mM ZnCl_2_ (Fig. 2c) or 300 µM CdCl_2_ (Fig. 2d), the Δ*zntA* mutant exhibited a strong reduction in CFU, and it showed large inhibition halos around disks with high concentrations of zinc and cadmium (Fig. 2e, f). In both assays, Δ*zntA* survival was rescued by the expression of *zntA* from a plasmid in the complemented strain (Fig. 2c, d, e, f), and the empty plasmid had no effect (Fig. 2e, f; Fig. S5). Taken together, these data indicate that the P_1B-2_-type ATPase ZntA is a key determinant of *C. violaceum* resistance against zinc and cadmium toxicity.

### Loss of *zntA* increases intracellular accumulation of zinc and cadmium

**Fig. 3:**
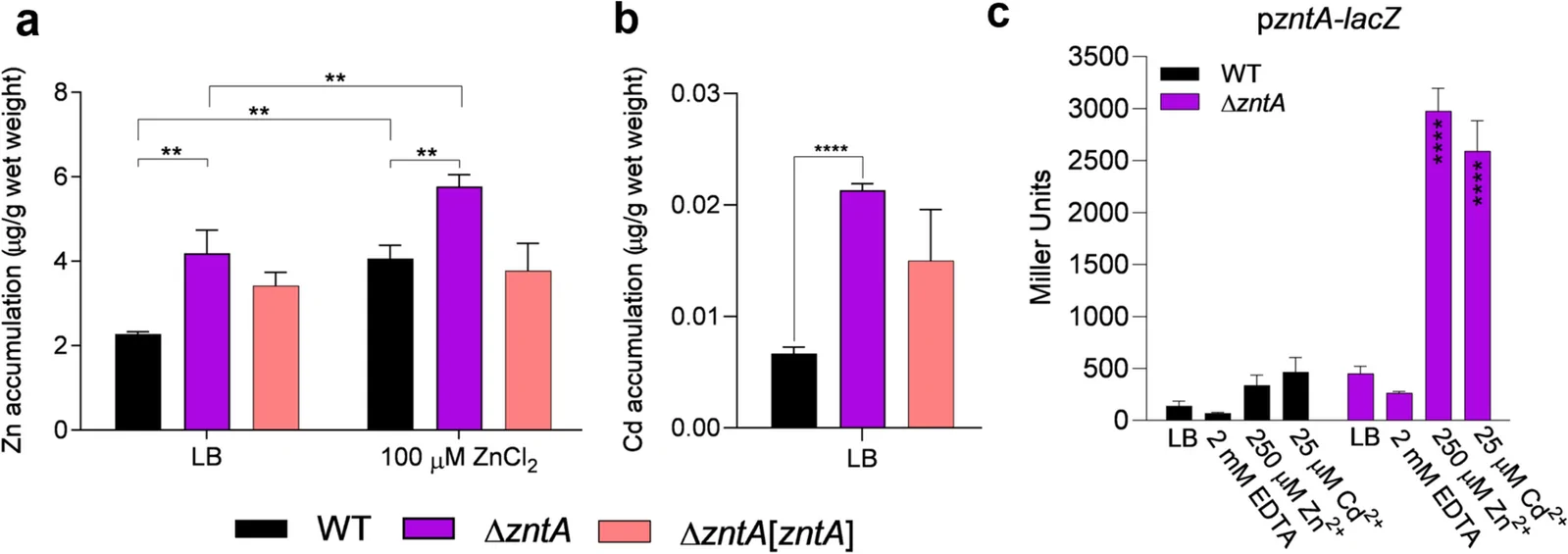
The Δ*zntA* mutant accumulates high levels of zinc and cadmium. **a** Intracellular levels of zinc in the indicated *C. violaceum* strains grown in LB or LB plus 100 μM ZnCl_2_.** b** Intracellular levels of cadmium in the indicated *C. violaceum* strains grown in LB. The bacterial cultures were grown until midlog growth phase, cells were pelleted by centrifugation, and metals were measured by ICP-MS. **c** *zntA* promoter activity is increased in the Δ*zntA* mutant strain. Cultures were grown in LB and treated or not with of 2 mM ethylenediaminetetraacetic acid (EDTA), 250 µM ZnCl_2_, or 25 µM CdCl_2_ for 45 min. β-galactosidase assays were performed using the indicated strains carrying the p*zntA-lacZ* fusion. Bars represent the mean ± SD of three independent biological replicates. Statistical significance was assessed by Two-Way ANOVA followed by Tukey’s multiple comparisons test. Vertical bars indicate comparisons with the control condition (Δ*zntA* strain in LB). **p < 0.01; ***p < 0.001; ****p < 0.0001; when not indicated, not significant

Given that ZntA is predicted to be a P_1B-2_-type ATPase and that a Δ*zntA* mutant displays high sensitivity to excess zinc and cadmium, it is hypothesized that this phenotype results from intracellular accumulation of these metals. To determine whether loss of ZntA leads to intracellular metal accumulation, inductively coupled plasma mass spectrometry (ICP-MS) was employed to analyze the levels of zinc and cadmium in *C. violaceum* WT, Δ*zntA* mutant, and Δ*zntA*[*zntA*] complemented strain grown in LB or LB plus 100 µM ZnCl_2_ (Fig. 3a, b). In LB, the Δ*zntA* mutant exhibited a twofold increase in zinc levels compared to that of the WT strain. Following ZnCl_2_ supplementation, zinc levels increased in all strains, but the Δ*zntA* mutant consistently maintained higher zinc levels compared to the WT strain. In the complemented strain, zinc levels dropped to those of the WT strain under zinc excess, supporting the role of ZntA in zinc efflux (Fig. 3a). A similar pattern was observed for cadmium. Even present in trace levels in LB, this toxic metal accumulated more in the Δ*zntA* mutant than in the WT strain with a partial reduction in the complemented strain (Fig. 3b), revealing that ZntA also contributes to cadmium efflux.

Since the *zntA* promoter responds specifically to elevated levels of zinc and cadmium (Fig. 1e), β-galactosidase activity assays were employed to evaluate the p*zntA-lacZ* transcriptional fusion in a Δ*zntA* mutant to infer indirectly whether zinc and cadmium accumulate intracellularly in this strain (Fig. 3c). In this case, low doses of the metals (LB with 250 µM ZnCl_2_ or 25 µM CdCl_2_) were used due to the sensitivity of Δ*zntA*. Upon treatment with zinc and cadmium, *zntA* expression was markedly higher in the Δ*zntA* mutant compared to that of the WT strain (Fig. 3c), indicating that the Δ*zntA* mutant accumulates higher levels of zinc and cadmium. Taken together, these results demonstrate the role of ZntA as a zinc and cadmium efflux pump that contributes to *C. violaceum* protection against intracellular accumulation and toxicity of these metals.

### *zntA* is positively regulated by ZntR in response to high levels of zinc and cadmium

**Fig. 4:**
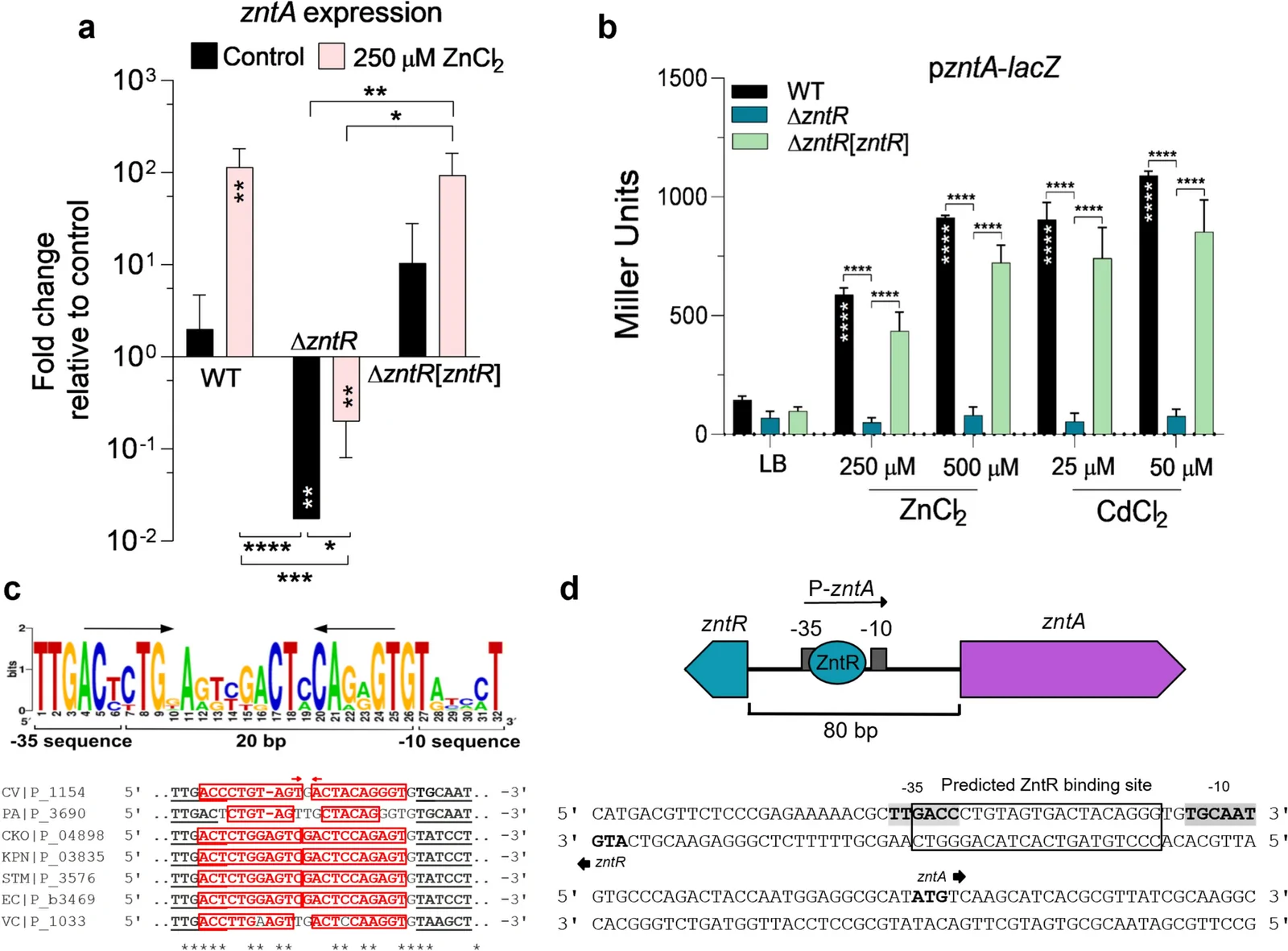
ZntR activates *zntA* in response to high levels of zinc and cadmium. **a** Reverse transcription quantitative real-time PCR (RT-qPCR) analysis of *zntA* expression. Total RNA was extracted from the *C. violaceum* indicated strains grown in LB (control) or LB plus 250 µM ZnCl_2_ for 15 min. Expression is shown as fold change relative to the control condition (WT strain in LB). **b** *zntA* promoter activity under different concentrations of zinc and cadmium. β-galactosidase assays were performed using the indicated strains carrying the P*zntA-lacZ* fusion grown in LB (control) or LB plus the indicated metals for 45 min. **a, b** Bars represent the mean ± SD of three biological replicates. Statistical analysis was performed using Two-Way ANOVA followed by Sidak’s multiple comparisons test. *p < 0.1; **p < 0.01; ***p < 0.001; ****p < 0.0001; when not indicated, not significant. Vertical bars indicate comparisons with the control condition (WT strain in LB). **c** Sequence logo and multiple sequence alignment of predicted ZntR-binding sites (ZntR boxes) in *zntA* promoters. Promoter regions of *zntA* from *Klebsiella pneumoniae* (KPN), *Pseudomonas aeruginosa* (PA), *Citrobacter koseri* (CKO), *Salmonella typhimurium* (STM), *Escherichia coli* (EC), and *Vibrio cholerae* (VC) were compared to the *C. violaceum* (CV) *zntA* promoter. Palindromic repeats are highlighted in red and indicated by arrows. Predicted − 35 and − 10 elements are underlined and bolded. Asterisks (*) indicate conserved nucleotides. **d** In silico analysis of the promoter region of *zntA* in *C. violaceum*. Identified elements include the − 35 and − 10 sequences, the start codon, and the putative ZntR-binding site. The position of the predicted ZntR box in relation to the *zntA* promoter is illustrated above the DNA sequence

In *C. violaceum*, the gene encoding the MerR family transcriptional regulator ZntR is located adjacent to *zntA* (Fig. 1a; Fig. S1b). To investigate the regulatory effect of ZntR on *zntA* expression, RT-qPCR and β-galactosidase activity assays were performed in *C. violaceum* WT, Δ*zntR* mutant, and Δ*zntR*[*zntR*] complemented strain in LB without or with zinc and cadmium excess (Fig. 4a, b). The RT-qPCR analysis revealed an approximately 100-fold increase in *zntA* expression in the WT strain upon exposure to excess zinc (Fig. 4a). Consistent with earlier findings (Fig. 1e), β-galactosidase assays confirmed that *zntA* expression in the WT strain increases proportionally as zinc and cadmium concentrations rise (Fig. 4b). In contrast, in both assays, *zntA* expression was markedly reduced in the Δ*zntR* mutant compared to the WT strain, even when cultures were treated with excess zinc or cadmium. The metal-induced *zntA* expression pattern was restored in the complemented strain (Fig. 4a, b). These results support the conclusion that ZntR functions as a transcriptional activator of *zntA* in response to high levels of zinc and cadmium.

To better understand the direct regulation of ZntR on the *zntA* gene, an in silico analysis in the *C. violaceum* genome was performed by searching for predicted ZntR-binding sites (ZntR boxes) of other bacteria, obtained from the RegPrecise database (Novichkov et al. 2013). By delimiting the search to the 80-base pair intergenic region containing the divergently transcribed promoters *zntA* and *zntR* of *C. violaceum* (Fig. 4c, d), a 21-nt palindromic DNA sequence (ACCCTGTAGTGACTACAGGGT) was identified. Multiple sequence alignment including more nucleotides around the predicted ZntR-binding sites revealed a high degree of conservation in the − 35 region among these promoters, whereas the − 10 region exhibited little conservation. The spacing between the − 35 and − 10 elements ranged from 19 to 20 base pairs, an arrangement typical of promoters regulated by ZntR and other MerR family regulators (Fang and Zhang 2022). Notably, all predicted ZntR-binding sites are palindromic DNA sequences (Fig. 4c). Based on these predictions, the putative ZntR-binding site is found between the − 35 and − 10 elements of *zntA* (Fig. 4d), which is consistent with ZntR activating *zntA* (Fig. 4a, b).

### A *zntR* mutant exhibits a zinc and cadmium sensitivity profile similar to the *zntA* mutant

**Fig. 5:**
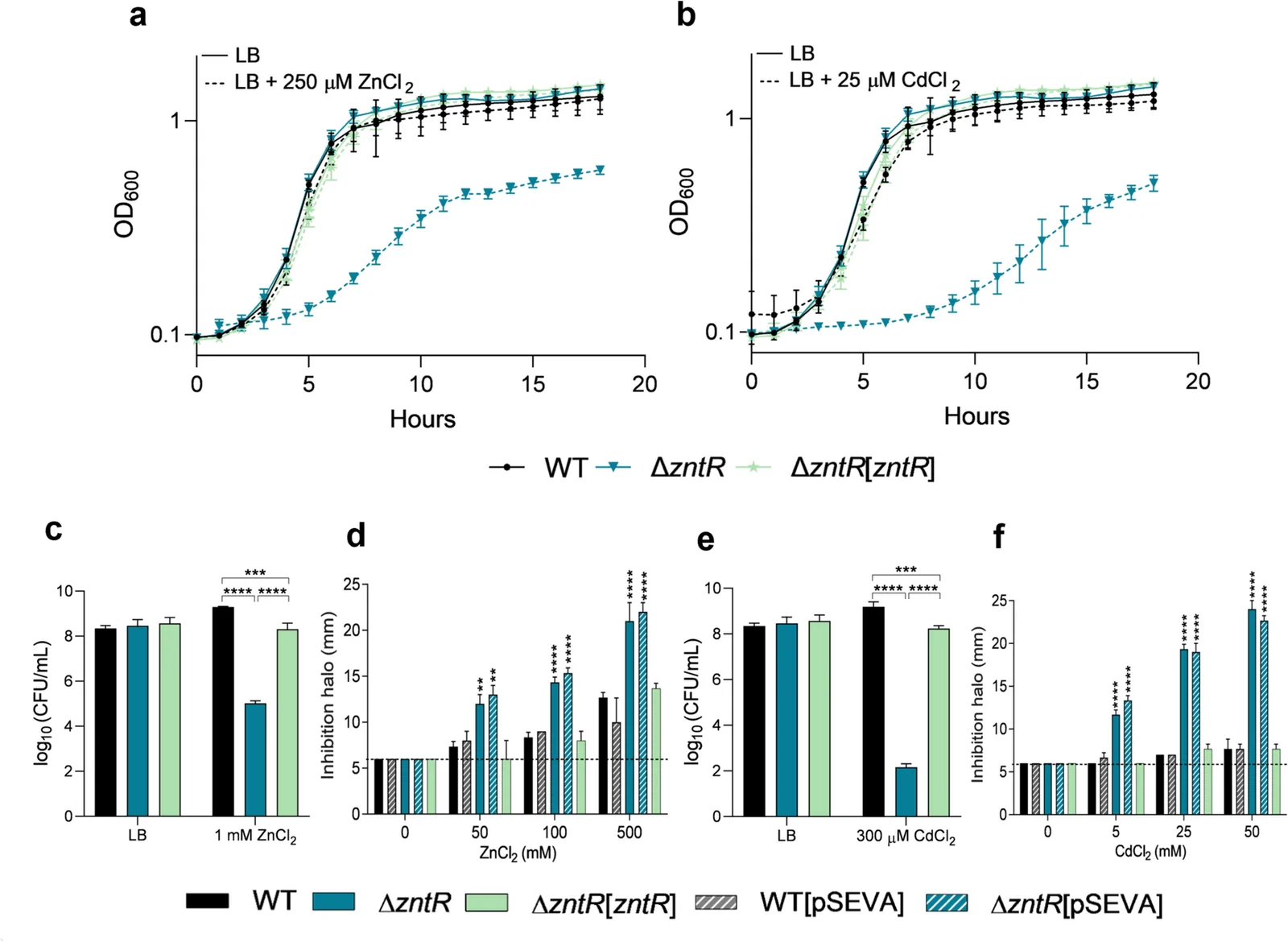
Sensitivity of the Δ*zntR* mutant to zinc and cadmium excess in *C. violaceum*. **a-b** Growth curves of the Δ*zntR* mutant in LB under zinc and cadmium stress. The indicated strains were grown in LB (solid lines) or LB plus ZnCl_2_ (**a**) or CdCl_2_ (**b**) (dashed lines). The OD_600nm_ was measured every 60 min over 18 h using a BioTek Epoch 2 microplate reader. Data represent the mean ± SD of three biological replicates. **c, e** Colony-forming unit (CFU/mL) assays of wild-type (WT), Δ*zntR* mutant, and complemented strains grown in LB with or without treatment of 1 mM ZnCl_2_ (**c**) or 300 µM CdCl_2_ (**e**). Midlog-phase cultures were serially diluted (undiluted to 10⁻⁷), and 10 µL of each dilution were spotted on LB plates for CFU counting. **d, f** Disk diffusion assays measuring the diameter of growth inhibition halos for the indicated strains exposed to ZnCl_2_ (**d**) or CdCl_2_ (**f**). The dashed line indicates disk diameter. Bars represent the mean ± SD of three independent biological experiments. Statistical significance was determined by Two-Way ANOVA with Tukey’s post hoc test. Vertical bars denote comparisons with the control condition (WT strain). **p < 0.01; ***p < 0.001; ****p < 0.0001; when not indicated, not significant

Considering that ZntR activates *zntA* expression (Fig. 4a, b), it was investigated whether deletion of *zntR* would impair *zntA*-mediated metal detoxification, increasing sensitivity to zinc and cadmium. To evaluate this, phenotypic characterization of a Δ*zntR* mutant was performed by growth curve, CFU, and disk diffusion assays (Fig. 5; Fig. S6). Growth curve assays revealed that the Δ*zntR* mutant was more sensitive than the WT strain to excess zinc and cadmium, with strong growth impairment in 250 µM ZnCl_2_ (Fig. 5a) and 25 µM CdCl_2_ (Fig. 5b), and complete growth inhibition at higher concentrations of these metals (Fig. S6). The phenotypes of zinc and cadmium sensitivity were recovered in the Δ*zntR*[*zntR*] complemented strain (Fig. 5a, b; Fig. S6). Consistent with these findings, the CFU assays revealed that the Δ*zntR* mutant exhibited reduced viability after 2 h of exposure to 1 mM ZnCl_2_ (Fig. 5c) or to 300 µM CdCl_2_ (Fig. 5e), whereas both the WT and complemented strains maintained CFU levels comparable to untreated controls. Similarly, in the disk diffusion assays, the Δ*zntR* mutant showed larger zones of inhibition than the WT and complemented strain under excess zinc (Fig. 5d) and cadmium (Fig. 5f). For both metals, the Δ*zntR* mutant displayed a milder sensitivity phenotype compared to the Δ*zntA* mutant (Fig. 2; Fig. 5; Fig. S5). One possible explanation for this phenotypic difference is that the Δ*zntR* mutant still expresses *zntA* at basal levels sufficient to provide partial protection. Taken together, these findings demonstrate that ZntR confers protection against zinc and cadmium toxicity in *C. violaceum*, most likely by acting as an activator of the P_1B-2_-type ATPase ZntA.

### *D. discoideum* as a model to study *C. violaceum* virulence

**Fig. 6:**
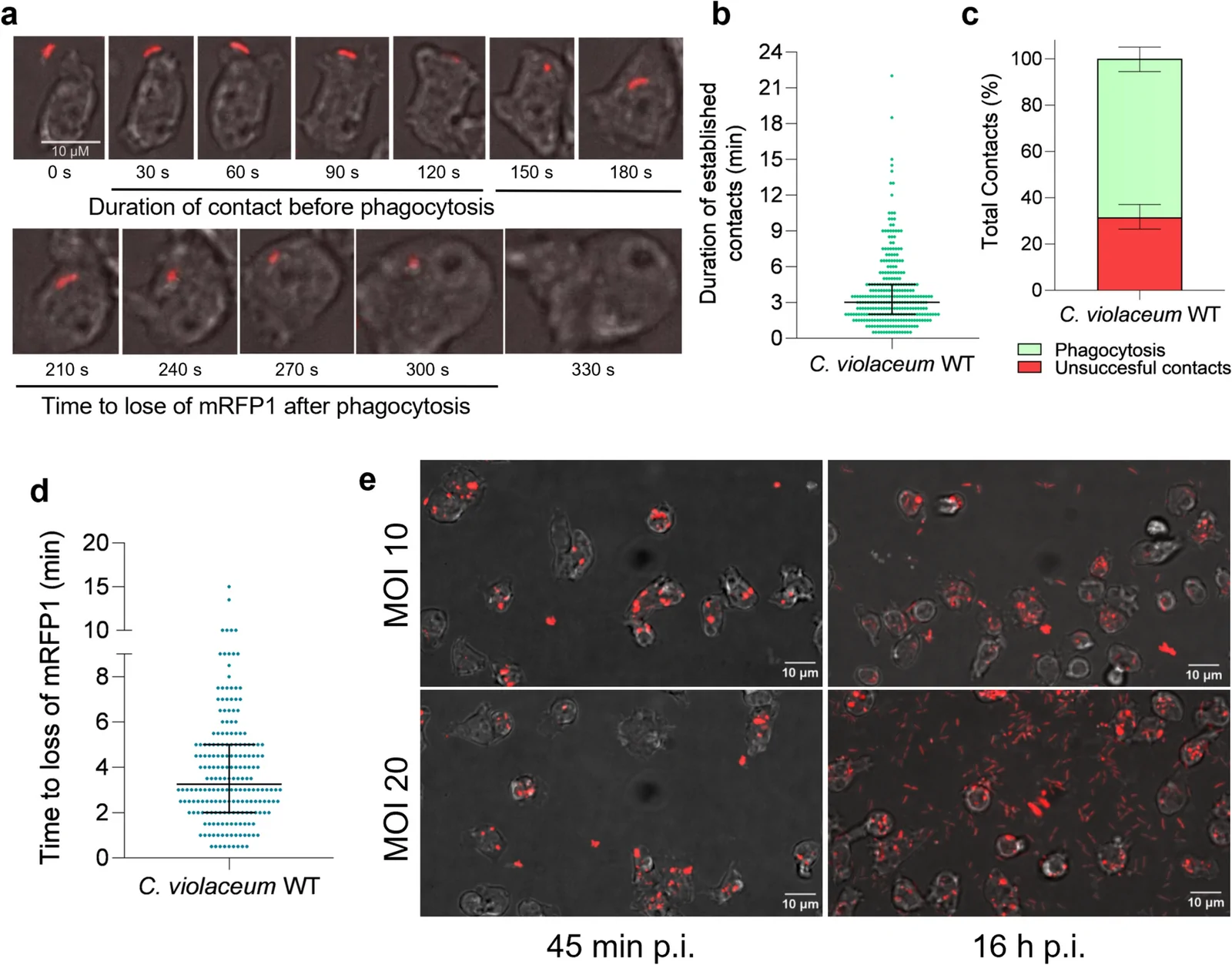
*D. discoideum* phagocytosis dynamics and intracellular killing of *C. violaceum*. **a** Representative time-lapse sequence illustrating how contact duration and intracellular killing were quantified. In this example, amoeba-bacterium contact begins at 30 seconds (s) and persists until 120 s, when phagocytosis occurs. The mRFP1 signal is lost at 300 s, corresponding to a killing time of 150 s. **b** Distribution of the duration of established contacts prior to phagocytosis (min). **c** Proportion of total contacts that resulted in successful phagocytosis versus unsuccessful contacts. Bars represent the mean ± SD. **d** Distribution of times to loss of the mRFP1 signal following phagocytosis (min). **e** Representative images of the infection of *D. discoideum* with *C. violaceum* at multiplicities of infection (MOIs) of 10 and 20, captured at 45 min and 16 h post-infection (p.i.). A total of 50 *D. discoideum* cells and 295 *C. violaceum* cells were analyzed. Black lines represent the median and error bars denote the interquartile range (b and c). Images were acquired by a spinning-disk confocal microscope. Scale bars, 10 μm. Imaging parameters: 20% laser power, 100 ms exposure time, 100 × magnification

The social amoeba *D. discoideum* has been widely used as a model to study host–pathogen interactions (Annesley and Fisher 2009; Froquet et al. 2009; Jauslin et al. 2021); however, to our knowledge, no studies have investigated its use with *C. violaceum*. The interaction between *C. violaceum* WT expressing mRFP1 and *D. discoideum* Ax2 (Ka) was examined using live-cell microscopy assays (Fig. 6). Following the bacterial fate in a protocol of 1:1 encounters (Fig. 6a), it was found that most phagocytosis events were preceded by brief bacteria-amoeba contacts (Fig. 6b), followed by phagocytosis of the bacteria in 70% of the contacts (Fig. 6c). Once internalized, bacteria were rapidly killed, with losing the mRFP1 signal between 3 and 5 min after ingestion in most cases (Fig. 6d). In these assays, *D. discoideum* death was not observed. To examine the interaction under conditions in which *C. violaceum* overwhelms the amoebae, an infection assay by spinoculation was performed using MOIs of 10 and 20, allowing the synchronization of the encounters. After the centrifugation rounds, the unigested bacteria were washed off and the medium supplemented with 60 µg/mL gentamicin to prevent the extracellular replication (Fig. 6e). At 45 min post-infection (p.i.), more than 50% of *D. discoideum* cells were infected, and only a few extracellular bacteria remained under both MOI conditions. By 16 h p.i., large numbers of bacteria were observed inside multiple *D. discoideum* cells. This likely reflects replication of bacteria that survived antibiotic effect and/or amoebae predation. Under MOI 20 at 16 h p.i, some *D. discoideum* cells exhibited a rounded morphology indicative of stress (Fig. 6e). Together, these results demonstrate that *C. violaceum* displays limited resistance to phagocytosis and to killing at low density, but exhibits virulence when present in overwhelming excess.

### *zntA* confers *C. violaceum* resistance against zinc stress imposed by *D. discoideum*

**Fig. 7:**
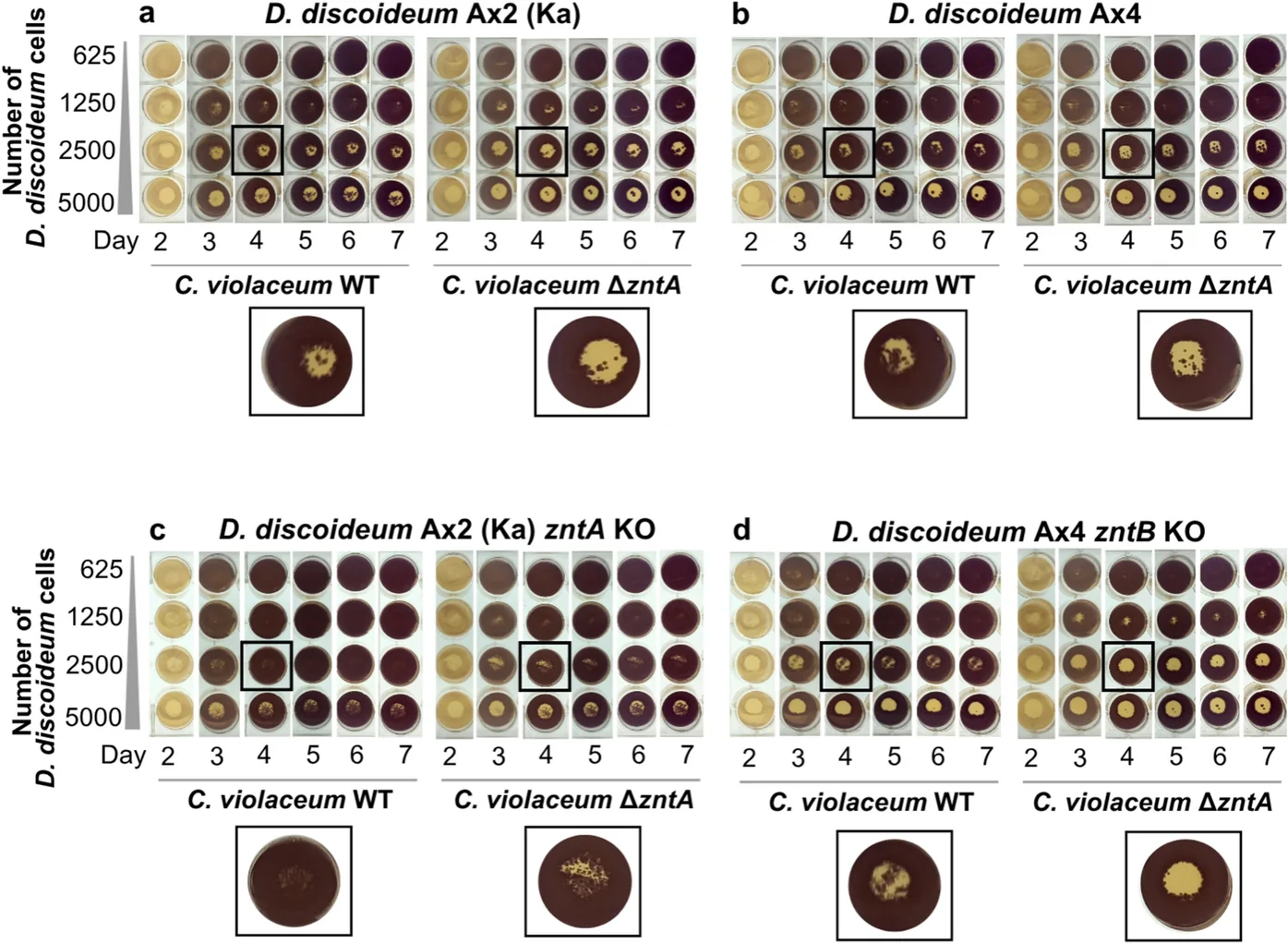
The *C. violaceum* Δ*zntA* mutant strain is more susceptible to predation by *D. discoideum zntA* and *zntB* knockout mutants. Images of a representative experiment of the growth of *D. discoideum* strains on lawns of *C. violaceum* WT and Δ*zntA* mutant. **a** *D. discoideum* Ax2 (Ka); **b** *D. discoideum* Ax4; **c** *D. discoideum* Ax2 (Ka) *zntA* KO; **d** *D. discoideum* Ax4 *zntB* KO cells. Different numbers of *D. discoideum* cells were spotted on the center of each well: 625, 1250, 2500, and 5000 cells. Plates were incubated at 22 °C, and images were captured daily over a 7-day period to observe the formation and expansion of plaques. Days two to seven are indicated in the figure. The plaques/halos within the squares (2,500 *D. discoideum* cells on day 4) are shown in the zoomed-in panels

To investigate whether ZntA contributes to *C. violaceum* protection against zinc intoxication in vivo, we sought to determine whether deletion of *zntA* alters *C. violaceum* susceptibility to bacterial clearance by *D. discoideum*, particularly under conditions where host intracellular zinc homeostasis is altered. To assess this, plaque assays were employed in which bacterial lawns of either WT or the Δ*zntA* mutant of *C. violaceum* were exposed to increasing numbers of *D. discoideum* cells, and evaluated the formation of plaques/halos over a period of 7 days (i.e. the area where the bacteria were cleared by amoeba) (Fig. 7). Two WT *D. discoideum* strains, Ax2 (Ka) and Ax4, were employed, which served as the genetic backgrounds for the construction of *zntA* and *zntB* knockout (KO) strains, respectively (Barisch et al. 2018; Hanna et al. 2021). *D. discoideum* DdZntA is the primary transporter of zinc to the contractile vacuole, and its deletion leads to an accumulation of this metal within zincosomes, phagosomes and the mycobacteria-containing vacuole. Therefore, the increased concentration of zinc inside *zntA* KO phagosomes promotes faster intracellular killing of *E. coli* and resistance of the amoebae to *M. marinum* infection. Otherwise, DdZntB acts as the main transporter involved in moving zinc from the cytosol to the phagolysosomal compartment, and its absence results in a lower lumenal zinc concentration. Amoeba DdZntA and DdZntB proteins belong to the ZnT (Zn^2+^ transporter Slc30a) family of zinc exporters (Barisch et al. 2018; Hanna et al. 2021).

In both Ax2 (Ka) and Ax4 amoebae laboratory strains, *C. violaceum* Δ*zntA* mutant exhibited a slightly higher sensitivity to amoeba compared to *C. violaceum* WT, indicated by the larger halos observed more clearly when 2,500 amoebae were added to lawns of the bacterial mutant strain (Fig. 7a, b). The *C. violaceum* Δ*zntA* susceptibility to amoeba was exacerbated when *D. discoideum zntA* or *zntB* KO cell lines were used (Fig. 7c, d), in which intracellular zinc homeostasis is altered (Barisch et al. 2018; Hanna et al. 2021). Whilst the knockout of *zntA* in *D. discoideum* conferred a protective effect to the C. *violaceum* WT and Δ*zntA* strains (i.e., reducing the diameter of the halos formed compared to WT amoebae) (Fig. 7a, c), in the assays with *D. discoideum zntB* KO, no effect on plaques were observed for *C. violaceum* WT, but the sensitivity of *C. violaceum* Δ*zntA* to clearance by amoeba was remarkably increased (Fig. 7b, d). These results indicate that the *C. violaceum* Δ*zntA* mutant is more sensitive to bacterial clearance by amoeba in a context of host zinc imbalance. Therefore, ZntA of *C. violaceum* plays a role in its protection against zinc intoxication in vivo, contributing to bacterial adaptation to metal stress imposed by *D. discoideum*.

## Discussion

Zinc is an essential trace element that acts as a cofactor for proteins involved in many biological processes. However, at elevated concentrations, zinc becomes toxic to bacteria by causing protein mismetallation, disrupting many biological pathways (Capdevila et al. 2016; Chandrangsu et al. 2017). The bacterial pathogen *C. violaceum* relies on the ZnuABC transporter to import zinc and sustain many zinc-dependent processes, including virulence in a mouse infection model (Santos et al. 2021), yet the mechanisms of zinc resistance remain poorly understood. In this work, we identified and characterized the P_1B-2_-type ATPase ZntA and its transcriptional activator ZntR as key determinants of zinc and cadmium sensing and resistance in *C. violaceum*. The findings demonstrate that ZntA provides an advantage to *C. violaceum* during interaction with *D. discoideum* strains exhibiting intracellular zinc dysregulation, highlighting the importance of zinc in bacterial host interactions.

The BlastP search revealed seven P-type ATPase proteins from *C. violaceum* showing high similarity with *E. coli* ZntA. However, the following evidence were provided that the *C. violaceum* gene CV_1154 encodes a ZntA P-type ATPase specific for efflux and protection against zinc and cadmium: (i) the *C. violaceum* ZntA has conserved signature amino acids of the P_1B-2_-type ATPase subfamily (Fig. 1), whose members are typically involved in zinc and cadmium efflux (Argüello 2003; Argüello et al. 2011; Smith et al. 2014); (ii) the expression of *zntA* increased under excess of zinc and cadmium (Fig. 1), consistent with the metal-responsive regulation of *zntA* observed in other bacteria (Rensing et al. 1997; Ducret et al. 2020; Zheng et al. 2024); (iii) the *C. violaceum* Δ*zntA* mutant showed high sensitivity to zinc and cadmium (Fig. 2; Fig. S4) and increased intracellular accumulation of these metals (Fig. 3), in agreement with studies on ZntA of *E. coli*, *Klebsiella pneumoniae*, *Agrobacterium tumefaciens*, *Brucella ovis*, and *Vibrio parahaemolyticus* (Rensing et al. 1997; Chaoprasid et al. 2015; Maunders et al. 2022; Tartilán-Choya et al. 2024; Zheng et al. 2024). The metal specificity of *C. violaceum* ZntA was evident as increased expression of *zntA* and susceptibility of the Δ*zntA* mutant occurred only under excess of zinc and cadmium among several tested metals (Fig. 1e; Fig. S3). For nickel, the Δ*zntA* mutant displayed even a slightly enhanced growth (Fig. S3c) likely due to cross upregulation of non-specific efflux systems. This result contrasts with that seen in *V. parahaemolyticus*, in which a Δ*zntA* mutant is sensitive to nickel toxicity (Zheng et al. 2024). Our findings expand current knowledge of metal toxicity in *C. violaceum*, which has previously been limited to studies on cytochrome *bd*-mediated zinc resistance (Batista et al. 2025) and cadmium interference with quorum-sensing signaling (Thornhill et al. 2017).

The gene CV_1153, located adjacent to *zntA*, was characterized as the MerR family transcriptional regulator ZntR. The results indicate that ZntR functions as a metal sensor that protects *C. violaceum* against zinc and cadmium toxicity (Fig. 5) by activating *zntA* expression in response to high levels of these metals (Fig. 4). A similar regulatory mechanism has been described in *E. coli*, *Cupriavidus metallidurans*, *V. parahaemolyticus*, and *A. tumefaciens* (Brocklehurst et al. 1999; Chaoprasid et al. 2015; Schulz et al. 2021; Zheng et al. 2024). Interestingly, ZntR from *Staphylococcus aureus* acts as a repressor of *zntA* (Singh et al. 1999). In silico analysis of the *zntA* promoter region from several bacteria revealed a conserved ZntR box located between the -35 and the -10 promoter elements, which are spaced by a 19–20 bp sequence (Fig. 4c), an arrangement typical of promoters activated by MerR-family regulators (Brocklehurst et al. 1999; Brown et al. 2003; Philips et al. 2015; Fang and Zhang 2022; Zheng et al. 2024). These predictions fit with a working model in which activation of *C. violaceum zntA* occurs upon binding of holo-ZntR to the ZntR box, realigning the -35 and -10 elements of the *zntA* promoter to favor RNA polymerase recruitment and transcription initiation (Fig. 4d).

The role of ZntA in vivo was investigated in an interaction model between *C. violaceum* and the social amoeba *D. discoideum*. Typically, lawns of non-pathogenic bacteria are efficiently cleared by *D. discoideum*, resulting in the progressive formation and expansion of plaques over time, even when amoebae are initially seeded at low densities (Froquet et al. 2009). By the fifth day of co-culture, *D. discoideum* had consumed most of the bacterial lawn and, in response to nutrient depletion, initiated fruiting body formation (Annesley and Fisher 2009). In contrast, when a small number of *D. discoideum* cells were exposed to *C. violaceum*, plaque formation was not observed (Fig. 7), suggesting resistance to clearance or inhibition of intracellular digestion in bacterial excess (Fig. 6e), a behavior typical of pathogenic bacteria.

The profiles of plaques were similar when *C. violaceum* WT and Δ*zntA* strains interacted with WT amoebae (Fig. 7a, b). However, when interacting with the *D. discoideum zntA* or *zntB* knockout strains that have perturbed intracellular zinc allocation, the Δ*zntA* mutant showed increased susceptibility to predation (Fig. 7c, d), suggesting that *C. violaceum* ZntA plays a role in zinc detoxification within intracellular environments with dysregulated zinc homeostasis. It has been reported that in the context of *M. marinum* infection, ZntA-deficient amoebae accumulate zinc in zincosomes and phagosomes, while ZntB-deficient amoebae exhibit elevated zinc in cytosol (Barisch et al. 2018; Hanna et al. 2021). Although the precise intracellular niche of *C. violaceum* inside *D. discoideum* remains to be determined, our results suggest that the zinc-detoxifying role of ZntA is important for *C. violaceum* during interaction with this host. The mycobacterial P-type ATPase CtpC confers bacterial protection against zinc intoxication triggered by amoeba and human macrophages (Botella et al. 2011; Hanna et al. 2021). Considering the essential role of a zinc uptake transporter for *C. violaceum* virulence in mice (Santos et al. 2021), future studies will be required to determine whether ZntA-mediated zinc detoxification is also relevant for *C. violaceum* infection in mammalian cells.

## Supplementary Information

Below is the link to the electronic supplementary material.

Supplementary file1 (PDF 956 KB)

## Data Availability

All data supporting the findings of this study are available within the paper and its Supplementary Information.

## References

[CR1] Andreini C, Banci L, Bertini I, Rosato A (2006) Zinc through the three domains of life. J Proteome Res 5:3173–3178. 10.1021/pr0603699 17081069 10.1021/pr0603699

[CR2] Annesley SJ, Fisher PR (2009) *Dictyostelium discoideum*: a model for many reasons. Mol Cell Biochem 329:73–91. 10.1007/s11010-009-0111-8 19387798 10.1007/s11010-009-0111-8

[CR3] Argüello J (2003) Identification of ion-selectivity determinants in heavy-metal transport P1B-type ATPases. J Membr Biol 195:93–108. 10.1007/s00232-003-2048-2 14692449 10.1007/s00232-003-2048-2

[CR4] Argüello JM, González-Guerrero M, Raimunda D (2011) Bacterial transition metal P(1B)-ATPases: transport mechanism and roles in virulence. Biochemistry 50:9940–9949. 10.1021/bi201418k 21999638 10.1021/bi201418k PMC3224801

[CR5] Barisch C, Kalinina V, Lefrançois LH, Appiah J, López-Jiménez AT, Soldati T (2018) Localization of all four ZnT zinc transporters in *Dictyostelium* and impact of ZntA and ZntB knockout on bacteria killing. J Cell Sci 131:jcs222000. 10.1242/jcs.222000 30404827 10.1242/jcs.222000

[CR6] Batista JH, da Silva Neto JF (2017) *Chromobacterium violaceum* pathogenicity: updates and insights from genome sequencing of novel *Chromobacterium* species. Front Microbiol 8:2213. 10.3389/fmicb.2017.02213 29176969 10.3389/fmicb.2017.02213 PMC5686120

[CR7] Batista BB, Santos RERS, Ricci-Azevedo R, da Silva Neto JF (2019) Production and uptake of distinct endogenous catecholate-type siderophores are required for iron acquisition and virulence in *Chromobacterium violaceum*. Infect Immun 87:e00577-19. 10.1128/IAI.00577-19 31570563 10.1128/IAI.00577-19 PMC6867865

[CR8] Batista BB, Will WR, de Lima VM, Fang FC, da Silva Neto JF (2025) A cytochrome *bd* repressed by a MarR family regulator confers resistance to metals, nitric oxide, sulfide, and cyanide in *Chromobacterium violaceum*. Appl Environ Microbiol 91:e0236024. 10.1128/aem.02360-24 39853125 10.1128/aem.02360-24 PMC11837568

[CR9] Bayer-Santos E, Cenens W, Matsuyama BY et al (2019) The opportunistic pathogen *Stenotrophomonas maltophilia* utilizes a type IV secretion system for interbacterial killing. PLoS Pathog 15:e1007651. 10.1371/journal.ppat.1007651 31513674 10.1371/journal.ppat.1007651 PMC6759196

[CR10] Begg SL, Eijkelkamp BA, Luo Z, Couñago RM, Morey JR, Maher MJ, Ong CL, McEwan AG, Kobe B, O’Mara ML, Paton JC, McDevitt CA (2015) Dysregulation of transition metal ion homeostasis is the molecular basis for cadmium toxicity in *Streptococcus pneumoniae*. Nat Commun 6:6418. 10.1038/ncomms7418 25731976 10.1038/ncomms7418 PMC4366526

[CR11] Botella H, Peyron P, Levillain F, Poincloux R et al (2011) Mycobacterial p(1)-type ATPases mediate resistance to zinc poisoning in human macrophages. Cell Host Microbe 10:248–259. 10.1016/j.chom.2011.08.006 21925112 10.1016/j.chom.2011.08.006 PMC3221041

[CR12] Brocklehurst KR, Hobman JL, Lawley B, Blank L, Marshall SJ, Brown NL, Morby AP (1999) ZntR is a Zn(II)-responsive MerR-like transcriptional regulator of *zntA* in *Escherichia coli*. Mol Microbiol 31:893–902. 10.1046/j.1365-2958.1999.01229.x 10048032 10.1046/j.1365-2958.1999.01229.x

[CR13] Brown NL, Stoyanov JV, Kidd SP, Hobman JL (2003) The MerR family of transcriptional regulators. FEMS Microbiol Rev 27:145–163. 10.1016/S0168-6445(03)00051-2 12829265 10.1016/S0168-6445(03)00051-2

[CR14] Capdevila DA, Wang J, Giedroc DP (2016) Bacterial strategies to maintain zinc metallostasis at the host-pathogen interface. J Biol Chem 291:20858–20868. 10.1074/jbc.R116.742023 27462080 10.1074/jbc.R116.742023 PMC5076499

[CR15] Chandrangsu P, Helmann JD (2016) Intracellular Zn(II) intoxication leads to dysregulation of the PerR regulon resulting in heme toxicity in *Bacillus subtilis*. PLoS Genet 12:e1006515. 10.1371/journal.pgen.1006515 27935957 10.1371/journal.pgen.1006515 PMC5189952

[CR16] Chandrangsu P, Rensing C, Helmann JD (2017) Metal homeostasis and resistance in bacteria. Nat Rev Microbiol 15:338–350. 10.1038/nrmicro.2017.15 28344348 10.1038/nrmicro.2017.15 PMC5963929

[CR17] Chaoprasid P, Nookabkaew S, Sukchawalit R, Mongkolsuk S (2015) Roles of *Agrobacterium tumefaciens* C58 ZntA and ZntB and the transcriptional regulator ZntR in controlling Cd2+/Zn2+/Co2+ resistance and the peroxide stress response. Microbiology 161:1730–1740. 10.1099/mic.0.000135 26296876 10.1099/mic.0.000135

[CR18] de Lima VM, Batista BB, da Silva Neto JF (2022) The regulatory protein ChuP connects heme and siderophore-mediated iron acquisition systems required for *Chromobacterium violaceum* virulence. Front Cell Infect Microbiol 12:873536. 10.3389/fcimb.2022.873536 35646721 10.3389/fcimb.2022.873536 PMC9131926

[CR19] Ducret V, Gonzalez MR, Leoni S, Valentini M, Perron K (2020) The CzcCBA efflux system requires the CadA P-type ATPase for timely expression upon zinc excess in *Pseudomonas aeruginosa*. Front Microbiol 11:911. 10.3389/fmicb.2020.00911 32477311 10.3389/fmicb.2020.00911 PMC7242495

[CR20] Eijkelkamp BA, Morey JR, Ween MP, Ong CL, McEwan AG, Paton JC, McDevitt CA (2014) Extracellular zinc competitively inhibits manganese uptake and compromises oxidative stress management in *Streptococcus pneumoniae*. PLoS ONE 9:e89427. 10.1371/journal.pone.0089427 24558498 10.1371/journal.pone.0089427 PMC3928430

[CR21] Fang C, Zhang Y (2022) Bacterial MerR family transcription regulators: activation by distortion. Acta Biochim Biophys Sin 54:25–36. 10.3724/abbs.2021003 35130613 10.3724/abbs.2021003 PMC9909328

[CR22] Froquet R, Lelong E, Marchetti A, Cosson P (2009) *Dictyostelium discoideum*: a model host to measure bacterial virulence. Nat Protoc 4:25–30. 10.1038/nprot.2008.212 19131953 10.1038/nprot.2008.212

[CR23] Gober JW, Shapiro L (1992) A developmentally regulated *Caulobacter* flagellar promoter is activated by 3’ enhancer and IHF binding elements. Mol Biol Cell 3:913–926. 10.1091/mbc.3.8.913 1392079 10.1091/mbc.3.8.913 PMC275648

[CR24] Hanahan D (1983) Studies on transformation of *Escherichia coli* with plasmids. J Mol Biol 166:557–580. 10.1016/s0022-2836(83)80284-8 6345791 10.1016/s0022-2836(83)80284-8

[CR25] Hanna N, Koliwer-Brandl H, Lefrançois LH, Kalinina V, Cardenal-Muñoz E, Appiah J, Leuba F, Gueho A, Hilbi H, Soldati T, Barisch C (2021) Zn2+ intoxication of *Mycobacterium marinum* during *Dictyostelium discoideum* infection is counteracted by induction of the pathogen Zn2+ exporter CtpC. Mbio 12:e01313-20. 10.1128/mBio.01313-20 33531393 10.1128/mBio.01313-20 PMC7858047

[CR26] Harvest CK, Abele TJ, Yu C et al (2023) An innate granuloma eradicates an environmental pathogen using Gsdmd and Nos2. Nat Commun 14:6686. 10.1038/s41467-023-42218-1 37865673 10.1038/s41467-023-42218-1 PMC10590453

[CR27] Hood MI, Skaar EP (2012) Nutritional immunity: transition metals at the pathogen-host interface. Nat Rev Microbiol 10:525–537. 10.1038/nrmicro2836 22796883 10.1038/nrmicro2836 PMC3875331

[CR28] Jauslin T, Lamrabet O, Crespo-Yañez X, Marchetti A, Ayadi I, Ifrid E, Guilhen C, Leippe M, Cosson P (2021) How phagocytic cells kill different bacteria: a quantitative analysis using *Dictyostelium discoideum*. Mbio 12(1):e03169-20. 10.1128/mBio.03169-20 33593980 10.1128/mBio.03169-20 PMC8545105

[CR29] Kapetanovic R, Bokil NJ, Achard ME et al (2016) *Salmonella* employs multiple mechanisms to subvert the TLR-inducible zinc-mediated antimicrobial response of human macrophages. FASEB J 30:1901–1912. 10.1096/fj.201500061 26839376 10.1096/fj.201500061

[CR30] Kolaj-Robin O, Russell D, Hayes KA, Pembroke JT, Soulimane T (2015) Cation Diffusion Facilitator family: structure and function. FEBS Lett 589:1283–1295. 10.1016/j.febslet.2015.04.007 25896018 10.1016/j.febslet.2015.04.007

[CR31] Maltez VI, Tubbs AL, Cook KD, Aachoui Y, Falcone EL, Holland SM, Whitmire JK, Miao EA (2015) Inflammasomes coordinate pyroptosis and natural killer cell cytotoxicity to clear infection by a ubiquitous environmental bacterium. Immunity 43:987–997. 10.1016/j.immuni.2015.10.010 26572063 10.1016/j.immuni.2015.10.010 PMC4654968

[CR32] Maunders EA, Ganio K, Hayes AJ, Neville SL, Davies MR, Strugnell RA, McDevitt CA, Tan A (2022) The role of ZntA in *Klebsiella pneumoniae* zinc homeostasis. Microbiol Spectr 10:e0177321. 10.1128/spectrum.01773-21 35019689 10.1128/spectrum.01773-21 PMC8754117

[CR33] Miki T, Iguchi M, Akiba K, Hosono M, Sobue T, Danbara H, Okada N (2010) *Chromobacterium* pathogenicity island 1 type III secretion system is a major virulence determinant for *Chromobacterium violaceum*-induced cell death in hepatocytes. Mol Microbiol 77:855–872. 10.1111/j.1365-2958.2010.07248.x 20545857 10.1111/j.1365-2958.2010.07248.x

[CR34] Neville SL, Eijkelkamp BA, Lothian A, Paton JC, Roberts BR, Rosch JW, McDevitt CA (2020) Cadmium stress dictates central carbon flux and alters membrane composition in *Streptococcus pneumoniae*. Commun Biol 3:694. 10.1038/s42003-020-01417-y 33214631 10.1038/s42003-020-01417-y PMC7678824

[CR35] Novichkov PS, Kazakov AE, Ravcheev DA, Leyn SA, Kovaleva GY, Sutormin RA, Kazanov MD, Riehl W, Arkin AP, Dubchak I, Rodionov DA (2013) RegPrecise 3.0--A resource for genome-scale exploration of transcriptional regulation in bacteria. BMC Genomics 14:745. 10.1186/1471-2164-14-745 24175918 10.1186/1471-2164-14-745 PMC3840689

[CR36] Pan Y, Zou J, Zhang K, Wang X, Ma Q, Mei L, Li Y, Pan Y (2023) ZccE, a P-type ATPase contributing to biofilm formation and competitiveness in *Streptococcus mutans*. Mol Oral Microbiol 38:198–211. 10.1111/omi.12405 36622758 10.1111/omi.12405

[CR37] Philips SJ, Canalizo-Hernandez M, Yildirim I, Schatz GC, Mondragón A, O’Halloran TV (2015) Allosteric transcriptional regulation via changes in the overall topology of the core promoter. Science 349:877–881. 10.1126/science.aaa9809 26293965 10.1126/science.aaa9809 PMC4617686

[CR38] Remick KA, Helmann JD (2023) The elements of life: a biocentric tour of the periodic table. Adv Microb Physiol 82:1–127. 10.1016/bs.ampbs.2022.11.001 36948652 10.1016/bs.ampbs.2022.11.001 PMC10727122

[CR39] Rensing C, Mitra B, Rosen BP (1997) The *zntA* gene of *Escherichia coli* encodes a Zn(II)-translocating P-type ATPase. Proc Natl Acad Sci USA 94:14326–14331. 10.1073/pnas.94.26.14326 9405611 10.1073/pnas.94.26.14326 PMC24962

[CR40] Rosenzweig AC, Argüello JM (2012) Toward a molecular understanding of metal transport by P(1B)-type ATPases. Curr Top Membr 69:113–136. 10.1016/B978-0-12-394390-3.00005-7 23046649 10.1016/B978-0-12-394390-3.00005-7 PMC3509741

[CR41] Santos RERS, Batista BB, da Silva Neto JF (2020) Ferric uptake regulator Fur coordinates siderophore production and defense against iron toxicity and oxidative stress and contributes to virulence in *Chromobacterium violaceum*. Appl Environ Microbiol 86:e01620-20. 10.1128/AEM.01620-20 32859594 10.1128/AEM.01620-20 PMC7580550

[CR42] Santos RERS, da Silva Júnior WP, Harrison S, Skaar EP, Chazin WJ, da Silva Neto JF (2021) The zinc transporter ZnuABC is critical for the virulence of *Chromobacterium violaceum* and contributes to diverse zinc-dependent physiological processes. Infect Immun 89:e0031121. 10.1128/IAI.00311-21 34370507 10.1128/IAI.00311-21 PMC8519291

[CR43] Schulz V, Schmidt-Vogler C, Strohmeyer P, Weber S, Kleemann D, Nies DH, Herzberg M (2021) Behind the shield of Czc: ZntR controls expression of the gene for the zinc-exporting P-type ATPase ZntA in *Cupriavidus metallidurans*. J Bacteriol 203:e00052-21. 10.1128/JB.00052-21 33685972 10.1128/JB.00052-21 PMC8117531

[CR44] Silva-Rocha R, Martínez-García E, Calles B, Chavarría M et al (2013) The standard European vector architecture (SEVA): a coherent platform for the analysis and deployment of complex prokaryotic phenotypes. Nucleic Acids Res. 10.1093/nar/gks1119 23180763 10.1093/nar/gks1119 PMC3531073

[CR45] Simon R, Prieffer U, Puhler A (1983) A broad host range mobilization system for in vivo genetic engineering: transposon mutagenesis in Gram-negative bacteria. Biotechnol 1:784–790. 10.1038/NBT1183-784

[CR46] Singh VK, Xiong A, Usgaard TR, Chakrabarti S, Deora R, Misra TK, Jayaswal RK (1999) ZntR is an autoregulatory protein and negatively regulates the chromosomal zinc resistance operon *znt* of *Staphylococcus aureus*. Mol Microbiol 33:200–207. 10.1046/j.1365-2958.1999.01466.x 10411736 10.1046/j.1365-2958.1999.01466.x

[CR47] Sitsel O, Grønberg C, Autzen HE, Wang K, Meloni G, Nissen P, Gourdon P (2015) Structure and function of Cu(I)- and Zn(II)-ATPases. Biochemistry 54:5673–5683. 10.1021/acs.biochem.5b00512 26132333 10.1021/acs.biochem.5b00512

[CR48] Smith AT, Smith KP, Rosenzweig AC (2014) Diversity of the metal-transporting P1B-type ATPases. J Biol Inorg Chem 19:947–960. 10.1007/s00775-014-1129-2 24729073 10.1007/s00775-014-1129-2 PMC4119550

[CR49] Stocks CJ, Phan MD, Achard MES, Nhu NTK, Condon ND, Gawthorne JA, Lo AW, Peters KM, McEwan AG, Kapetanovic R, Schembri MA, Sweet MJ (2019) Uropathogenic *Escherichia coli* employs both evasion and resistance to subvert innate immune-mediated zinc toxicity for dissemination. Proc Natl Acad Sci U S A 116:6341–6350. 10.1073/pnas.1820870116 30846555 10.1073/pnas.1820870116 PMC6442554

[CR50] Tartilán-Choya B, Tejedor C, Conde-Álvarez R, Muñoz PM, Vizcaíno N (2024) Characterization of three predicted zinc exporters in *Brucella ovis* identifies ZntR-ZntA as a powerful zinc and cadmium efflux system not required for virulence and unveils pathogenic Brucellae heterogeneity in zinc homeostasis. Front Vet Sci 10:1323500. 10.3389/fvets.2023.1323500 38260206 10.3389/fvets.2023.1323500 PMC10800456

[CR51] Thornhill SG, Kumar M, Vega LM, McLean RJC (2017) Cadmium ion inhibition of quorum signaling in *Chromobacterium violaceum*. Microbiology 163:1429–1435. 10.1099/mic.0.000531 28895513 10.1099/mic.0.000531

[CR52] Vasconcelos ATR, Almeida DF, Hungria M et al (2003) The complete genome sequence of *Chromobacterium violaceum* reveals remarkable and exploitable bacterial adaptability. Proc Natl Acad Sci U S A 100:11660–11665. 10.1073/pnas.1832124100 14500782 10.1073/pnas.1832124100 PMC208814

[CR53] Wang K, Sitsel O, Meloni G, Autzen HE, Andersson M, Klymchuk T, Nielsen AM, Rees DC, Nissen P, Gourdon P (2014) Structure and mechanism of Zn2+-transporting P-type ATPases. Nature 514:518–522. 10.1038/nature13618 25132545 10.1038/nature13618 PMC4259247

[CR54] Zheng C, Zhai Y, Qiu J, Wang M, Xu Z, Chen X, Zhou X, Jiao X (2024) ZntA maintains zinc and cadmium homeostasis and promotes oxidative stress resistance and virulence in *Vibrio parahaemolyticus*. Gut Microbes 16:2327377. 10.1080/19490976.2024.2327377 38466137 10.1080/19490976.2024.2327377 PMC10936601

