# Supplementary material for: The genes zntA and zntR encoding a P-type ATPase and its activator confer zinc and cadmium resistance in Chromobacterium violaceum
Source: Biometals. 2026 May 22;39(5):1747–65. doi: 10.1007/s10534-026-00828-4 (PMC13518485; doi:10.1007/s10534-026-00828-4)
Supplement: Supplementary file 1 [file 10534_2026_828_MOESM1_ESM.pdf]

The genes *zntA* and *zntR* encoding a P-type ATPase and its activator confer zinc and cadmium resistance in *Chromobacterium violaceum*

Carolina Panosso Schuindt[1]; Bianca Bontempi Batista[1]; Lucas Cesetti[2]; Sergio Pereira Lima Neto[1]; Thierry Soldati[2]; José Freire da Silva Neto[1*]

[1]Departamento de Biologia Celular e Molecular e Bioagentes Patogênicos, Faculdade de Medicina de Ribeirão Preto, Universidade de São Paulo, Ribeirão Preto, Brazil.

[2]Department of Biochemistry, University of Geneva, Sciences II, CH-1211 Genève-4, Geneva, Switzerland.

*For correspondence: José F da Silva Neto, jfsneto@usp.br

BioMetals

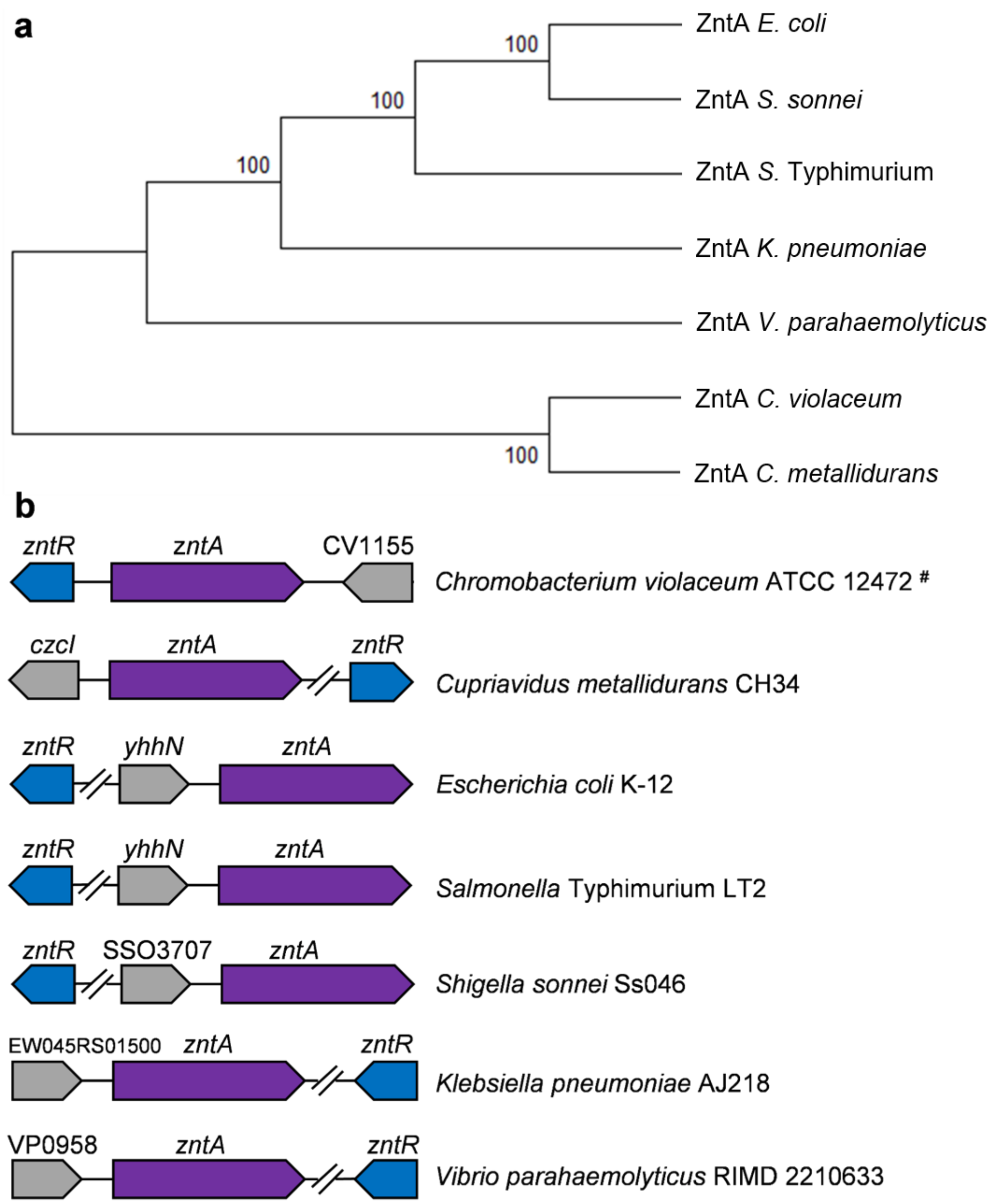

Fig. S1. Phylogenetic tree and genomic context of *zntA*. a Characterized ZntA proteins from selected bacteria were aligned using the CLUSTALW program. The phylogenetic tree was constructed using the neighbor-joining method based on the CLUSTALW alignment with the software MEGA (https://megasoftware.net/). **b** The schematic depicts the chromosomal region surrounding the *zntA* gene. The *zntR* gene is located either adjacent to (*C. violaceum*) or very distant from (other bacteria shown) *zntA*. # The adjacent and divergent arrangement of *zntA/zntR* is found in most genomes of *Chromobacterium* species as analyzed with IMG/M tools (https://img.jgi.doe.gov/cgi-bin/m/main.cgi).

a

Condition LB + [$ZnCl_2$]	Lagtime (min ± SD)	Doubling Time (min ± SD)
0 µM	189 ± 7	90 ± 6
50 µM	210 ± 5	81 ± 2
100 µM	219 ± 4	82 ± 3
250 µM	210 ± 6	83 ± 3
500 µM	209 ± 6	88 ± 2
1000 µM	211 ± 18	104 ± 14
2000 µM	265 ±5	131 ± 10

b

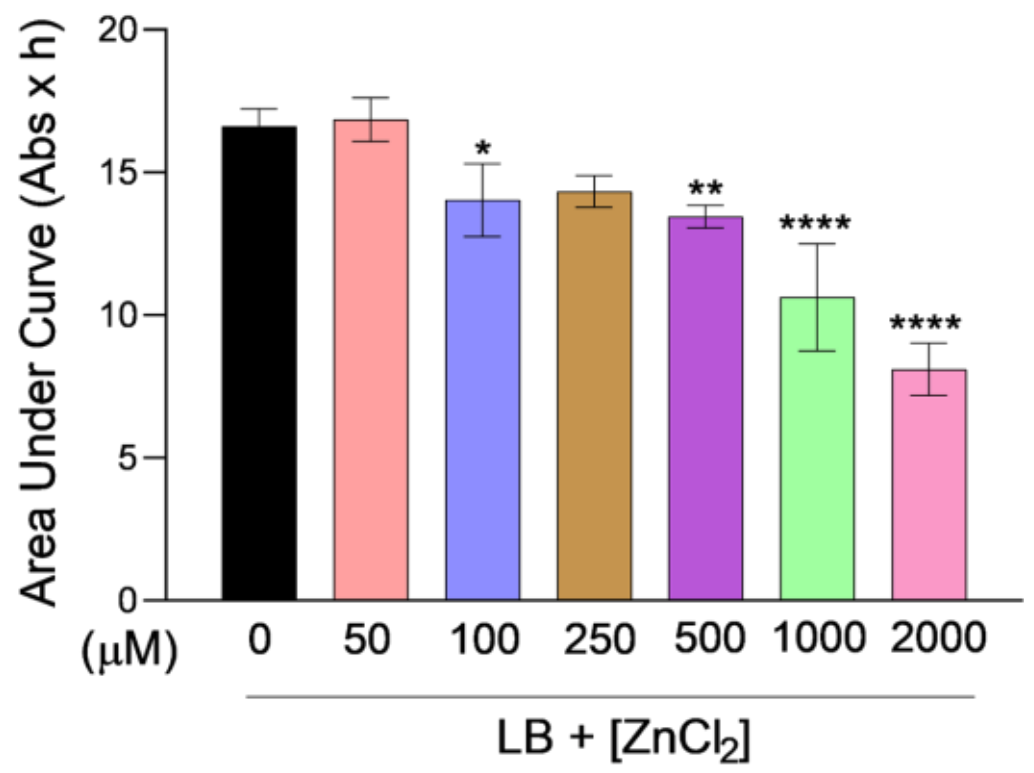

c

Condition LB + [$CdCl_2$]	Lagtime (min ± SD)	Doubling Time (min ± SD)
Control	189 ± 7	90 ± 6
10 µM	198 ± 12	81 ± 1
25 µM	234 ± 4	94 ± 2
100 µM	254 ± 4	103 ± 7
250 µM	230 ± 17	106 ± 7
500 µM	233 ± 6	113 ± 2
1000 µM	266 ± 31	117 ± 14

d

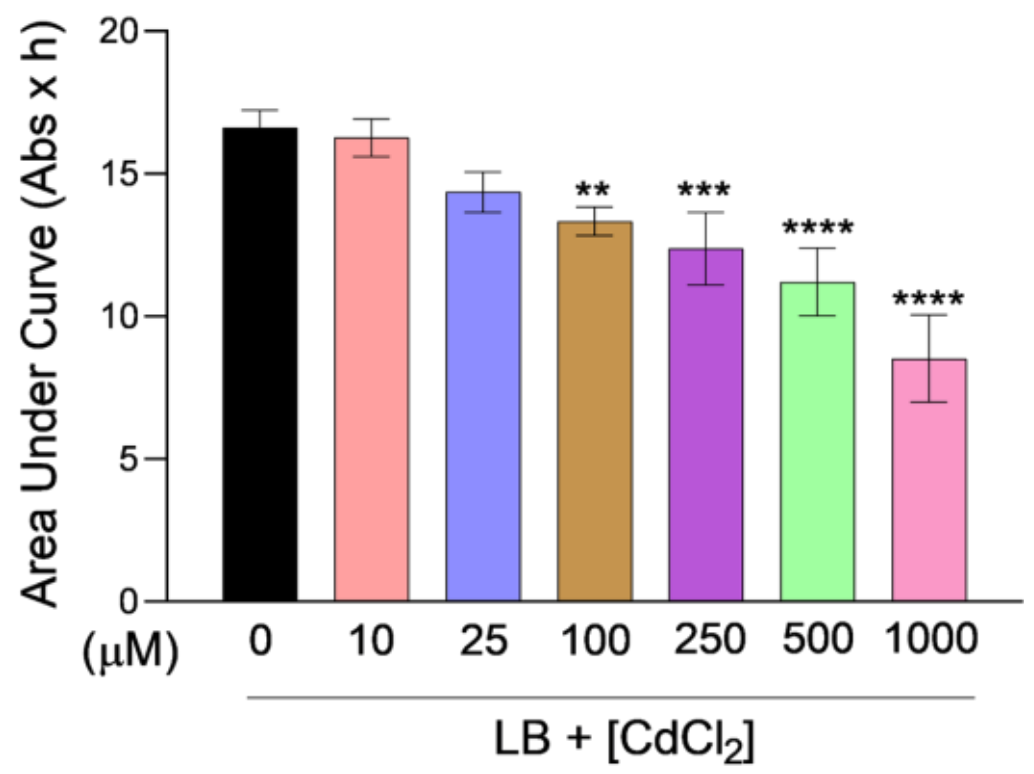

Fig. S2. Growth parameters of *C. violaceum* WT exposed to increasing concentrations of $ZnCl_2$ and $CdCl_2$. a Lag time and doubling time (min ± SD) of *C. violaceum* WT grown in LB supplemented with the indicated concentrations of $ZnCl_2$. **b** Area under the curve (AUC) corresponding to the growth curves treated with $ZnCl_2$. **c** Lag time and doubling time (min ± SD) of *C. violaceum* WT grown in LB supplemented with the indicated concentrations of $CdCl_2$. **d** Area under the curve (AUC) corresponding to the growth curves treated with $CdCl_2$. Data represent three biological replicates. *$p < 0.1$; **$p < 0.01$; ***$p < 0.001$; ****$p < 0.0001$.

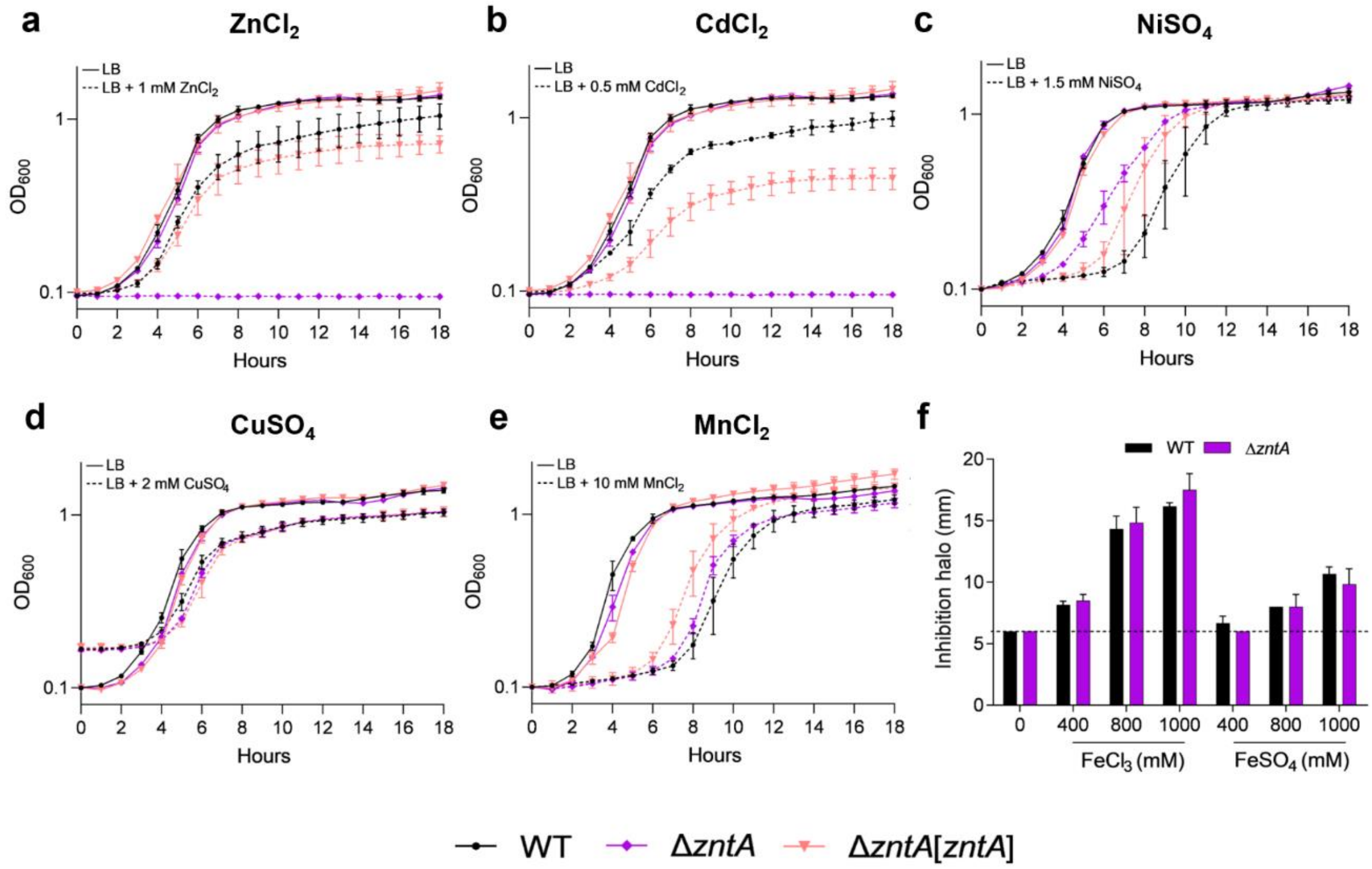

Fig. S3 The Δ*zntA* mutant strain shows sensibility only under zinc and cadmium excess. a-e. Growth curves of the Δ*zntA* mutant strain under treatment with $ZnCl_2$ (**a**), $CdCl_2$ (**b**), $NiSO_4$ (**c**), $CuSO_4$ (**d**), and $MnCl_2$ (**e**). Growth was measured using a BioTek Epoch 2 microplate reader, with OD_{600nm} readings taken every 60 minutes over 18 hours. Solid lines represent growth in LB medium, while dashed lines indicate the respective treatments. **f** Measurement of the growth inhibition halo diameter for the indicated strains in the disk diffusion assay with $FeCl_3$ and $FeSO_4$. The dashed line represents the size of the disk.

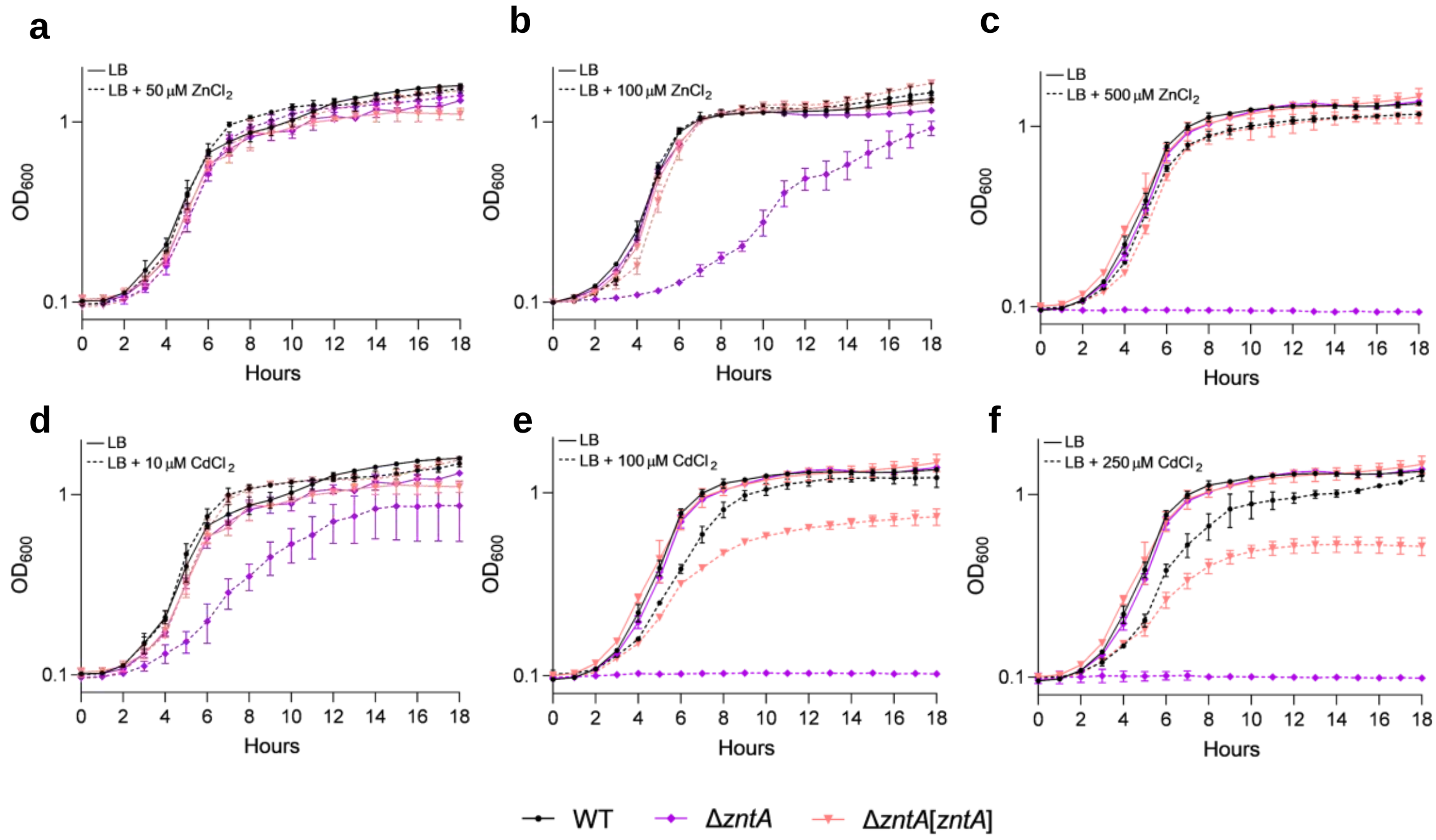

Fig. S4 Growth curves of Δ*zntA* mutant in LB under zinc and cadmium stress. The indicated strains were grown in LB (solid lines) or LB plus zinc or cadmium (dashed lines). **a-c** Increased concentrations of $ZnCl_2$. **d-f** Increased concentrations of $CdCl_2$. The OD_{600nm} was measured every 60 minutes over 18 hours using a BioTek Epoch 2 microplate reader. Data represent the mean ± SD of three biological replicates.

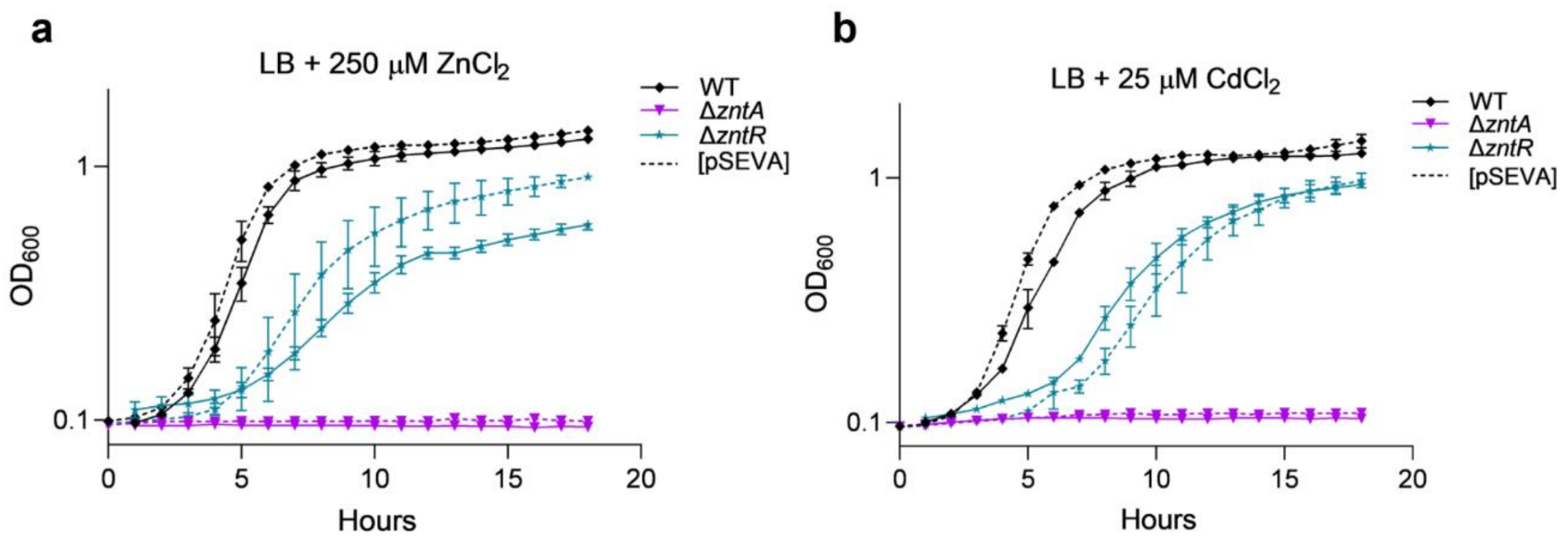

Fig. S5 Growth curves of the Δ*zntA* and Δ*zntR* mutant strains carrying the empty vector pSEVA. Solid lines represent the strains without vector, while dashed lines indicate the strains harboring the empty vector pSEVA. **a** Growth in LB supplemented with 250 µM $ZnCl_2$. **b** Growth in LB supplemented with 25 µM $CdCl_2$. Growth was measured using a BioTek Epoch 2 microplate reader, with OD_{600nm} readings taken every 60 minutes over 18 hours. Data from three biological replicates.

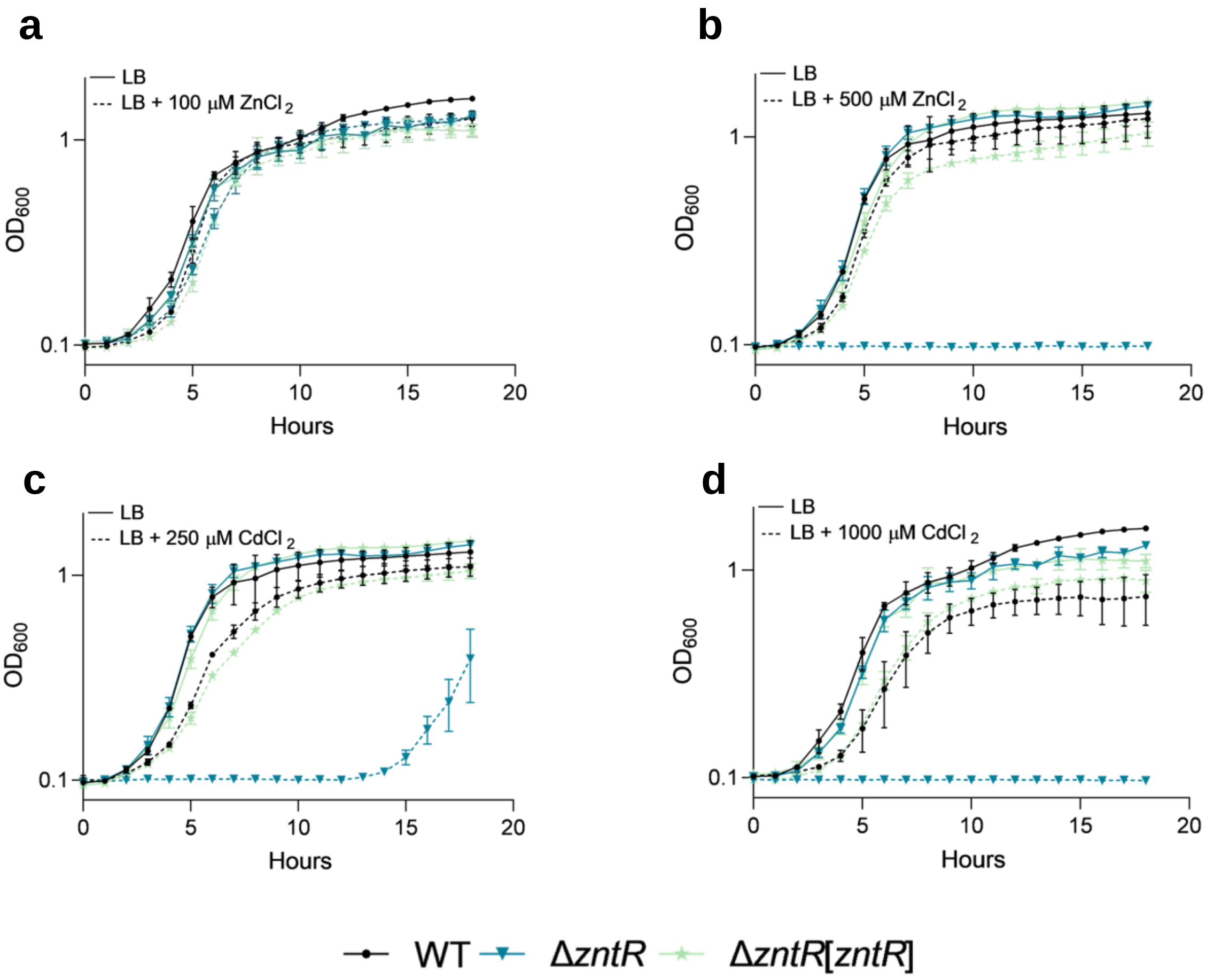

Fig. S6 Growth curves of Δ*zntR* mutant in LB under zinc and cadmium stress. The indicated strains were grown in LB (solid lines) or LB plus zinc or cadmium (dashed lines). **a-b** Increased concentrations of $ZnCl_2$. **c-d** Increased concentrations of $CdCl_2$. The OD_{600nm} was measured every 60 minutes over 18 hours using a BioTek Epoch 2 microplate reader. Data represent the mean ± SD of three biological replicates.

Table S1. Primers used in this work.

Primer name	Sequence (5’→3’)[a]	Description
**Construction of mutant strains**		
CV_1154 del1	TACCGGAAGCTTCCAGCAGCCGCAGAGCTTCC	Cloning of the CV_1154 (*zntA*) gene flanking region into pNPTS138 for mutagenesis (597 bp; HindIII/BamHI)
CV_1154 del2	TACCGGGGATCCATCATGCTTGTGGTCGTGCCC	
CV_1154 del3	TACCGGGGATCCCTGTGGATGGCGGTGTTCGC	Cloning of the CV_1154 (*zntA*) gene flanking region into pNPTS138 for mutagenesis (601 bp; BamHI/EcoRI)
CV_1154 del4	TACCGGGAATTCGATCCAGATCAGCCAGGGCG	
CV_1153 del1	TACCGGAAGCTTGGCGATGGCGCTCCTGAC	Cloning of the CV_1153 (*zntR*) gene flanking region into pNPTS138 for mutagenesis (602 bp, HindIII/BamHI)
CV_1153 del2	TACCGGGGATCCGGTTTCCACCTCGCAGCC	
CV_1153 del3	TACCGGGGATCCCAGGACTACGACCACGCG	Cloning of the CV_1153 (*zntR*) gene flanking region into pNPTS138 for mutagenesis (752 bp, BamHI/EcoRI)
CV_1153 del4	TACCGGGAATTCGAAGTATTACCTGAAGACGCG	
**Construction of complemented strains**		
CV_1154 Comp Fw	TACCGGGGTACCGCGCCAGTTCTCCGATCAAC	Cloning of the CV_1154 (*zntA*) gene into pSEVA221 for complementation (2762 bp, KpnI/BamHI)
CV_1154 Comp Rv	TACCGGGGATCCACACCTACGCCTGATGGCCG	
CV_1153 Comp Fw	TACCGGGGTACCGCGTCTGCCGCTCTCCGC	Cloning of the CV_1153 (*zntR*) gene into pSEVA221 for complementation (796 bp; KpnI/BamHI)
CV_1153 Comp Rv	TACCGGGGATCCCTGTAACGATTGTGTTGTGAG	
**β-galactosidase assay**		
CV_1154 del2	TACCGGGGATCCATCATGCTTGTGGTCGTGCCC	Cloning of upstream region of CV_1154 (*zntA*) gene into pRK*lacZ*290 for β-galactosidase assay (315 bp, BamHI/KpnI)
CV_1154 Comp Fw	TACCGGGGTACCGCGCCAGTTCTCCGATCAAC	
**RT-qPCR assay**		
CV_1154_qPCR_FW2	AGCGCTTCATCGATCGCTT	Expression of CV_1154 (*zntA*) gene in the RT-qPCR (117 bp)
CV_1154_qPCR_RV2	TGTAGATCGCCTGGTGCCA	
CV_3376_qRT-PCR_Fw	GCGCTGATGTCGCTCTACTT	Expression of CV_3376 (*minD*) gene in the RT-qPCR (112 bp)
CV_3376_qRT-PCR_Rv	GCTTGGACTTGGACGACAGG	

[a] The underline sequences indicate sites for restriction enzymes.

Table S2. *C. violaceum* proteins showing high identity with *E. coli* ZntA.

Genome identifier	Uniprot	Blast Evalue	Identity	Subgroups of P-type ATPases
CV_1154 (ZntA)	Q7NYW8	1e-127	38.10%	P1B-2-subgroup
CV_1265	Q7NYK9	6e-107	37.70%	P1B-1-subgroup
CV_3217	Q7NT45	8e-73	33.46%	P1B-1-subgroup
CV_2042	Q7NWE8	1e-58	30.11%	P1B-1-subgroup
CV_2748	Q7NUF1	7e-54	34.34%	P1B-5-subgroup
CV_1598	Q7NXM8	8e-41	30.68%	1A P-Type ATPase
CV_3100	Q7NTF6	4e-16	30.64%	3B ATPases
